# Laboratory conditions matter: social housing in male and female laboratory mice in a multicenter study approach

**DOI:** 10.1038/s41684-026-01805-7

**Published:** 2026-10-13

**Authors:** Anne S. Mallien, Anna Munk, Anne Rix, Miriam A. Vogt, Natascha Pfeiffer, Verena Buchecker, Renee Girbig, Sarah Neunecker, Laura Becker, Monika Franz, Jasmin Baier, Christiane Brandwein, André Bleich, Rupert Palme, René Tolba, Heidrun Potschka, Fabian Kießling, Sabine Chourbaji, Steven R. Talbot, Peter Gass

**Affiliations:** 1 Research Group Animal Models in Psychiatry, Department of Psychiatry and Psychotherapy, Medical Faculty Mannheim, Central Institute of Mental Health, Heidelberg University, Heidelberg, Germany; 2 German Center for Mental Health (DZPG), Partner Site Mannheim, Heidelberg, Ulm Germany; 3 Institute of Pharmacology, Toxicology, and Pharmacy, Ludwig-Maximilians-Universität, Munich, Germany; 4 Institute for Experimental Molecular Imaging, Uniklinik RWTH Aachen, Aachen, Germany; 5 Interfaculty Biomedical Research Facility, University of Heidelberg, Heidelberg, Germany; 6 Hannover Medical School, Institute for Laboratory Animal Science, Hannover, Germany; 7 Department of Biological Sciences and Pathobiology, University of Veterinary Medicine, Vienna, Austria; 8 Institute for Laboratory Animal Science and Experimental Surgery, RWTH Aachen University, Aachen, Germany

**Keywords:** Animal behaviour, Lab life

## Abstract

Despite numerous studies and literature reviews, the social housing of laboratory mice is still under debate. Aggression and social stress can lead to physiological and behavioral alterations, especially in male mice, whereas single housing is a potential source of discomfort, particularly in female mice. This multicenter study aimed to assess the impact of single versus group housing on severity in both sexes and to examine whether a general recommendation could be made. C57BL/6NRj mice from the same litters of a commercial breeder were housed either singly or in groups of four across four different laboratory sites. Here we assessed fecal corticosterone metabolite concentration, aggressive behavior, wound scores and observed behavioral readouts on locomotion, stress coping in the forced swim test, burrowing, nesting and anxiety. Differences due to social housing conditions were not very pronounced. Instead, we found that, despite harmonization efforts, the differences in outcomes across the four laboratory sites were substantial. Furthermore, the individuality of the mice appears to have an additional influence on the results. These findings suggest that optimal housing is not generalizable but depends on the specific laboratory conditions. Beyond meeting the minimum housing standards as set by legislation, we should actively embrace refinement as a guiding principle and strive to create housing conditions that not only meet scientific needs but also continuously improve the welfare of the animals entrusted to our care.

## Abstract (web summary)

This study compares single and group housing in male and female laboratory mice across four research sites, finding that site-specific conditions and individual variability have a greater impact than housing type.

## Main

Mice are still the most commonly used model organism in research^1^. They are often selected for qualities that make them particularly well suited for that purpose: there is a large body of knowledge about their genetics, physiology, anatomy and biology. To the current standard, they need little space owing to their size, have a relatively short life span and a high reproductive cycle. In addition, laboratory mice are easy to handle when trained correctly.

Wild mice generally live in commensal populations of polygamous family groups, with one male and several females and their offspring. The size of groups depends on the food supply and other resources^2,3^. Young, sexually mature males try to occupy territories, for example, by aggressively and sometimes fatally challenging territory holders or by solitary migration to find a new territory^4^. Habitats with sufficient resources allow for the tolerance of subdominant male mice in the territory of a less aggressive dominant male^4^.

This complex social network cannot be translated into the laboratory, as it would unnecessarily produce offspring, which is unethical. Thus, artificial sex-specific housing is the standard condition used in experiments, outside of breeding projects. This raises the question of how to house male and female mice, as finding the ideal husbandry system is critical for welfare, ethical and scientific reasons. The ETS123 Appendix A (European Council 2006)^5^ mandates keeping mice in stable and harmonious groups. This is often difficult to achieve in practice, as male animals are prone to aggression. Although the harms and benefits of group and single housing are known^6,7^, the field appears to remain divided based on the laboratory-specific experiences.

The German Society of Laboratory Animal Science (GV-SOLAS) states: “After reaching a certain age (depending on the strain), single housing of male mice is preferable to group housing with other males. The occurrence of bite wounds must not be the sole criterion here, because housing-related stress and anxiety states without any physical injury must also be avoided as far as possible. […] It is emphasized here that single housing does not mean isolation. Visual, audible, and olfactory contact remains intact.”^8^ Kappel et al.^6^ reviewed the problem of group and single housing in male mice and emphasized that “any situation is highly context dependent according to several factors including strain, age, social position, life experiences and housing and husbandry protocols.” This is further supported by the recommendations of the Swedish 3Rs Center^9^, which advises on how to circumvent and manage single housing owing to aggression in male mice.

A 2025 preprint challenges the prioritization of preferred group housing for male mice^10^, suggesting that under certain laboratory conditions, male mice may show indicators consistent with a preference for single housing. However, such findings must be interpreted with caution, as both single housing and same-sex group housing in standard laboratory cages represent artificial conditions that do not reflect the natural social organization of wild mice. In the wild, male mice typically live in complex social structures involving females and offspring, and social dynamics are shaped by environmental resources and the availability of space. Therefore, it remains unclear to what extent either housing condition can be considered more appropriate, and both may be associated with welfare impairments depending on the context. However, previous work by Van Loo et al.^11^ demonstrated that male mice show a preference for social contact with familiar conspecifics, despite the well-documented risk of aggression in group housing. Together, these findings highlight that both single and group housing represent compromises under laboratory conditions, and that neither can be considered universally optimal. The general recommendation for female mice is less controversial. They should be “basically housed in groups or at least pairs”^8^. Potential sex-specific preferences or suitability of housing conditions influence the experimental design of studies involving both sexes, representing a development in the field that contributes to more relevant and robust outcomes^12^. Severity is the degree to which negative states, such as pain, distress or impaired function, restrict an animal’s well-being. While well-being reflects the current physical and psychological state, welfare encompasses the overall quality of life, including both positive and negative experiences. Measuring physiological, biochemical and behavioral parameters enables the assessment of severity-associated changes as a restriction to the animals’ well-being. However, fully capturing the spectrum of welfare remains challenging.

This study compared the effects of two different housing conditions—single housing and group housing of four—on severity-associated changes in male and female mice by assessing physiological, biochemical and behavioral parameters. To ensure robust and generalizable conclusions, the study was conducted as a coordinated multicenter experiment in four laboratories within the DFG-funded research group FOR2591 (‘Severity assessment in animal-based research’)^13^. The aim was to compare the effects of different housing conditions and assess whether consistent patterns emerge across sexes and laboratory sites.

**Fig. 1:**
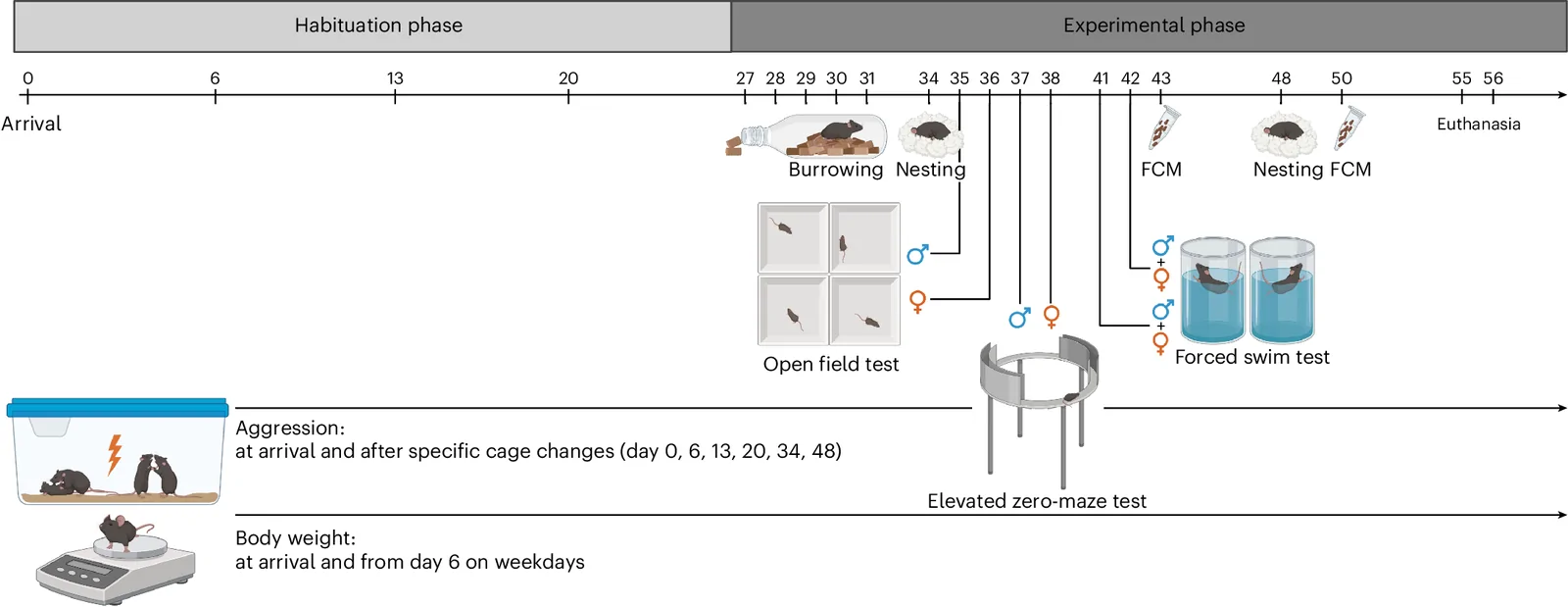
Overview of the experimental design. After an initial habituation and housing phase, experimental testing of behavioral tasks and feces collection started. Aggression and body weight were assessed during the habituation and experimental phase. Figure created in BioRender; Mallien, A. https://biorender.com/rtat7rh (2026).

Our study design (Fig. 1) aimed to balance harmonization of critical factors with preservation of the unique environmental conditions inherent to each participating laboratory. Rather than enforcing strict standardization, we allowed noncontrolled variables to reflect the real-world variability between sites. This approach maintains ecological validity and addresses the challenge of external reproducibility often faced in experimental research. The controlled factors for this study included the origin, age and sex of the mice, which were littermates distributed to four different laboratory sites at 5 weeks of age. Housing was determined to be either single-housed or in groups of four. Most of those factors have been shown to influence aggressive behavior^6,14,15^, which we wanted to exclude as confounding factors. We harmonized the daily routine, for example, circadian rhythm, testing time, handling time and procedure, test apparatus and housing and experimental protocols. Harmonizing parameters and scoring can limit variability and increase robustness^16^. All data were collected and centrally analyzed.

## Results

### Aggressive behavior was not consistent in all laboratories

**Fig. 2:**
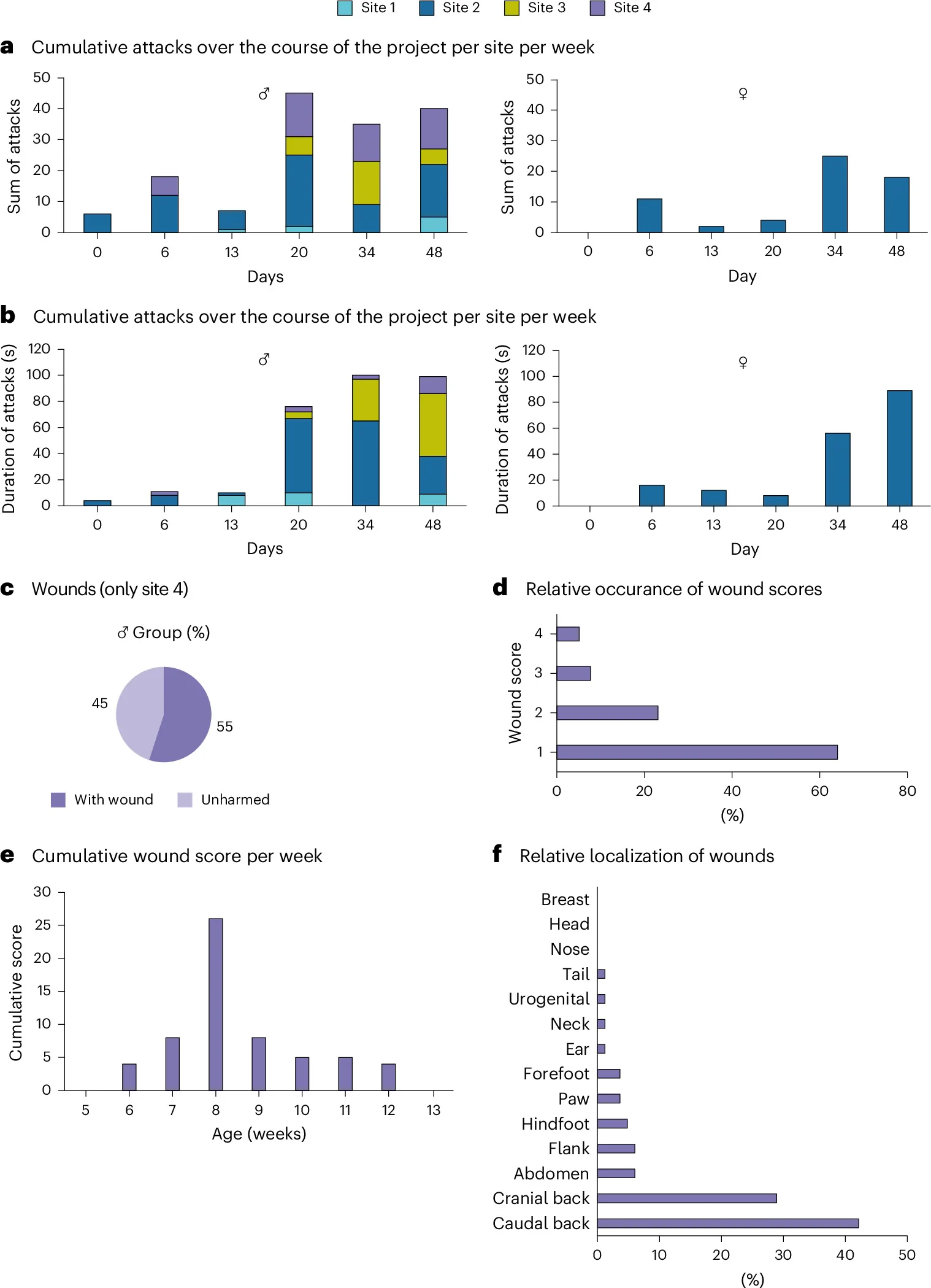
Observations on aggression and wound scoring. **a**, Total number of attacks per site in group-housed mice. **b**, Duration of attacks per site (s). Results in **a** and **b** are presented separately by sex, with males shown on the left and females on the right. **c**–**f**, Description of wounds in group-housed males at site 4. **c**, Proportion of males with and without wounds, showing that 45% of male mice were not affected by wounds. **d**, Percentage distribution of wound scores, with most detected wounds classified as score 1. **e**, Cumulative wound scoring over time. **f**, Anatomical locations of wounds. Data are presented as descriptive statistics, including absolute frequencies and percentages where indicated. As these analyses are descriptive in nature, no statistical hypothesis testing or *P* values were calculated. Sample sizes (*n*, male/female) were as follows: group-housed site 1: 20/20, site 2: 20/20, site 3: 20/20, site 4: 16/20.

In addition to the daily health inspection, we monitored aggressive behavior after cage changes. Group-housed male mice displayed more attacks than females at each laboratory site (Fig. 2a,b). Attacks were the most common at site 2, even showing attacks among female mice.

Although we detected a few short attacks in the 10 min after the cage change in site 4, it was the only laboratory to observe wounds in male mice. A total of 55% of group-housed male mice on site 4 showed wounds (Fig. 2c). The majority of assigned scores were wound score 1 (Fig. 2d). Unfortunately, we had to exclude one group cage from site 4 owing to intense aggression, resulting in one severely wounded male, who was euthanized immediately, and a too-high risk of further injuries in the remaining mice. Mice were separated on day 21. The wound scoring peaked at 8 weeks of age but improved over time (Fig. 2e). The back was the most affected localization in wounded males (Fig. 2f).

**Fig. 3:**
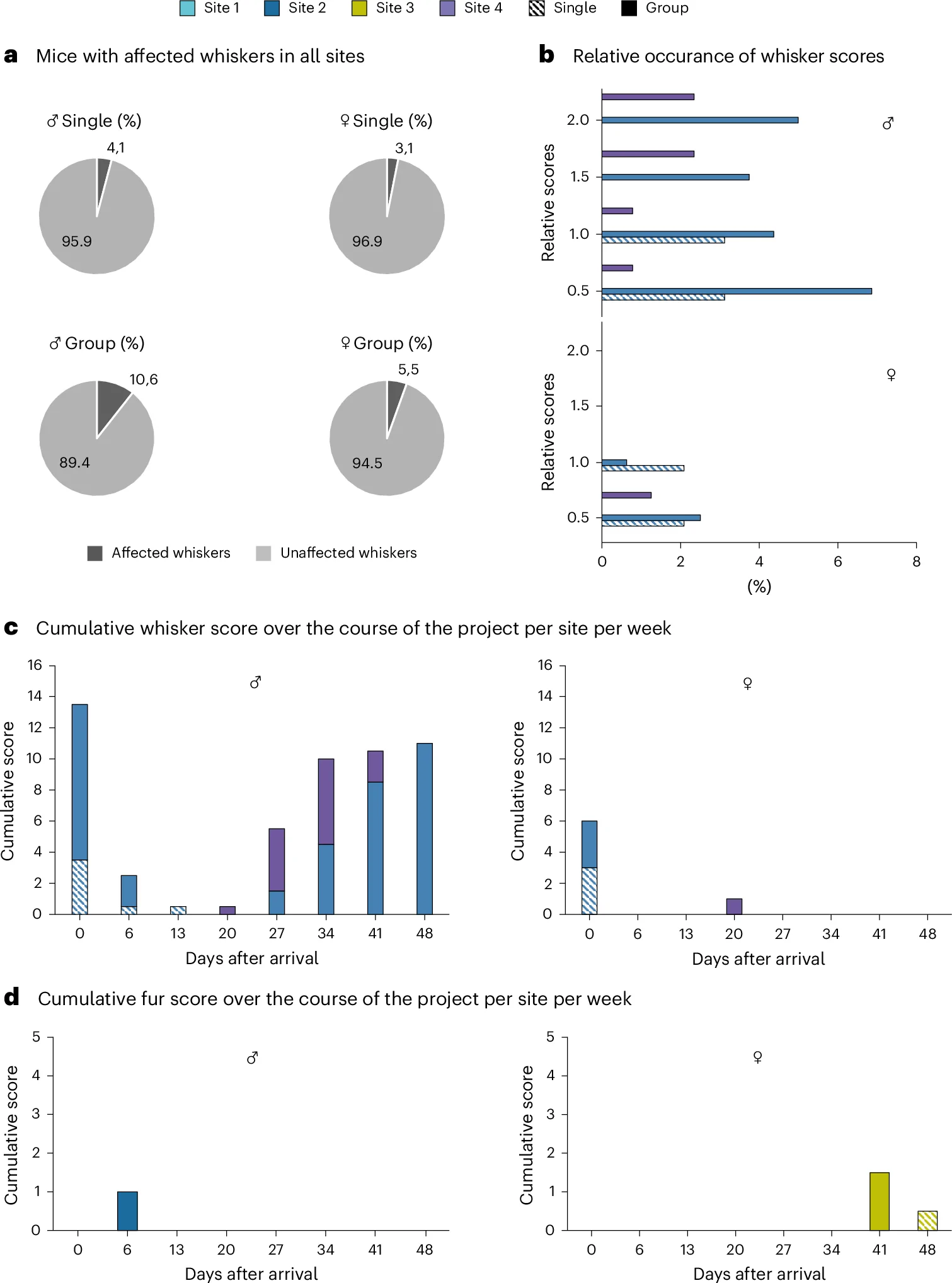
Whisker and fur score in all sites. **a**, Percentage of mice showing whisker impairments. **b**, Percentage of whisker scores detected in the mice. **c**, Cumulative whisker score over the course of the project per site per week. **d**, Cumulative fur score over the course of the project per site per week. Data are presented as descriptive statistics, including absolute frequencies and percentages where indicated. As these analyses are descriptive in nature, no statistical hypothesis testing or *P* values were calculated. Sample sizes (*n*, male/female) were as follows: single-housed site 1: 12/12, site 2: 12/12, site 3: 11/12, site 4: 12/12; group-housed—site 1: 20/20, site 2: 20/20, site 3: 20/20, site 4: 16/20.

Most mice did not show whisker impairment (Fig. 3a). The male group-housed mice were the most affected, with 10.6% impacted. Whisker impairments in female mice were less common and displayed lower scores, and only mice in sites 2 and 4 showed whisker alterations (Fig. 3b). The number of affected animals was the highest in the week of arrival and dropped before rising again (Fig. 3c). The counts for fur scores were very low (Fig. 3d).

### Burrowing behavior was not affected by social housing conditions

**Fig. 4:**
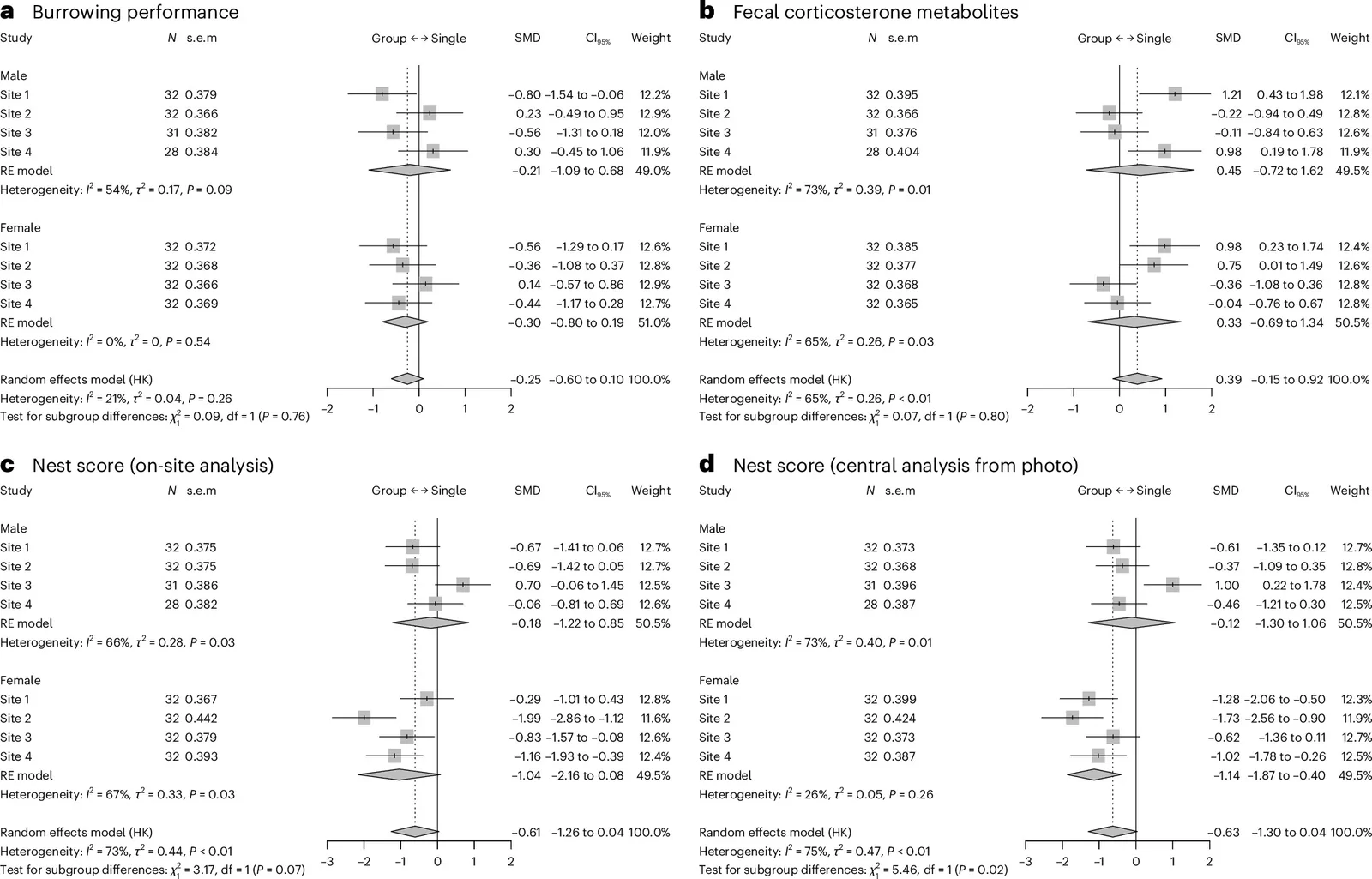
Home-cage-based analysis of severity-sensitive parameters. Forest plots represent the effect size for each laboratory. Positive effect sizes indicate a predominant outcome in single-housed mice. A negative effect size indicates an outcome in favor of the group-housed mice. Significant results do not cross the original line. **a**, Burrowing performance. **b**, Fecal corticosterone metabolite concentrations. **c**, Nest complexity scored by different experimenters at each site, which did not show significant differences between housing type. **d**, Nest complexity scored from photos by the same expert, which showed a mild effect of social housing condition. The nests in group-housed mice were more complex in this analysis. Forest plot showing SMDs with CI_95%_ for each included study. Sample sizes (*N*) are shown for each study in the corresponding columns of the forest plot. The numbers of male/female animals (*n*) were as follows: single-housed site 1: 12/12, site 2: 12/12, site 3: 11/12, site 4: 12/12; group-housed site 1: 20/20, site 2: 20/20, site 3: 20/20, site 4: 16/20. Square sizes are proportional to the study weights, and diamonds represent the pooled effect estimates obtained using a random-effects model with the Hartung–Knapp (HK) adjustment.

Although the group-housed animals burrowed more material than the single-housed in general, this was not significant (*g* = −0.25, 95% confidence interval (CI_95%_) −0.6 to 0.1, *t* = −1.71, *P* = 0.131) (Fig. 4a).

There were no significant differences between the sexes (*Q* = 0.09, df 1, *P* = 0.76). Heterogeneity between laboratories in the male studies did not reach significance (*Q* = 6.55, df 3, *P* = 0.09), although with only four laboratories, the test is underpowered and the estimates indicate moderate between-laboratory variability (*I*^2^ = 54%, *τ*^2^ = 0.17). In females, the heterogeneity was low (*I*^2^ = 0%), and no differences between the laboratories were detected (*Q* = 2.15, df 3, *τ*^2^ = 0, *P* = 0.54).

### Single housing led to increased or decreased fecal corticosterone metabolite concentrations dependent on site

Laboratory, but not housing, influenced the stress hormone release (*I*^2^ = 65%; *Q* = 63.23, df 3, *P* < 0.0001). No sex-specific effects were detected (*Q* = 0.07, df 1, *P* = 0.80). Significant effects were detected in site 1 for both sexes (*g* = 1.21, CI_95%_ 0.43 to 1.98, *P* = 0.002 and *g* = 0.98, CI_95%_ 0.23 to 1.74, *P* = 0.011), where single-housed mice showed higher fecal corticosterone metabolites (FCM) concentrations. The same effect was observed for the samples of males in site 4 (*g* = 0.98, CI_95%_ 0.19 to 1.78, *P* = 0.015) and females in site 2 (*g* = 0.75, CI_95%_ 0.01 to 1.49, *P* = 0.047), where FCM concentrations were significantly higher in single-housed mice (Fig. 4b).

### Nest complexity is sex but not housing dependent

Although the evaluation of the complexity of the nests has been highly harmonized, considerable heterogeneity was observed across studies (*I*^2^ = 73%, *Q* = 26.41, df 7, *P* = 0.0004 in the on-site scoring by multiple raters; Fig. 4c; *I*^2^ = 75%, *Q* = 28.01, df 7, *P* = 0.0002 in the scoring based on photos by a single rater; Fig. 4d), although this heterogeneity was not significantly attributable to laboratory as a subgroup factor (on site: *Q* = 1.93, df 3, *P* = 0.59; photo: *Q* = 1.78, df 3, *P* = 0.62).

Central photo-based analysis performed by a single trained expert on nest complexity indicated a trend toward higher scores in group-housed mice, although this did not reach statistical significance (*g* = −0.63, CI_95%_ −1.30 to 0.04, *t* = −2.24, *P* = 0.061). The results were driven by the female mice: female group-housed mice achieved significantly higher scores than single-housed females (*g* = −1.14, CI_95%_ −1.87 to −0.40, *t* = −4.94, *P* = 0.016), while no differences in nest scores were detected in male mice (*g* = −0.12, CI_95%_ −1.30 to 1.06, *t* = −0.32, *P* = 0.77).

Female mice in group housing tended to achieve higher nest scores than male mice (*Q* = 3.17, df 1, *P* = 0.075) in on-site screening: nests from females showed higher complexity (*g* = −1.04, CI_95%_ −2.16 to 0.08) than those in male cages (*g* = −0.18, CI_95%_ −1.22 to 0.85). In comparison, the analysis by photo detected a significant sex difference (*Q* = 5.46, df 1, *P* = 0.0195): the nest complexity was more pronounced in this data set (group-housed females, *g* = −1.14, CI_95%_ −1.87 to −0.40). The major differences between on-site and by-photo analysis were observed in female mice at site 1: the on-site data suggest no difference between the housing conditions (*g* = −0.29, CI_95%_ −1.01 to 0.43), while the blinded central analysis revealed a significantly more complex scoring of group-housed females, consistent across most sites (site 1: *g* = −1.28, CI_95%_ −2.06 to −0.50, *P* = 0.001; site 2: *g* = −1.73, CI_95%_ −2.56 to −0.90, *P* < 0.0001; site 4: *g* = −1.02, CI_95%_ −1.78 to −0.26, *P* = 0.009), with a similar but nonsignificant trend at site 3 (*g* = −0.62, CI_95%_ −1.36 to 0.11, *P* = 0.10). One major observation from on-site scoring was that nests were built using bedding material without necessarily shredding the cotton nestlet, but using it as a lid, which leads to lower scores.

### Housing effects on open field behavior were site dependent rather than consistent

**Fig. 5:**
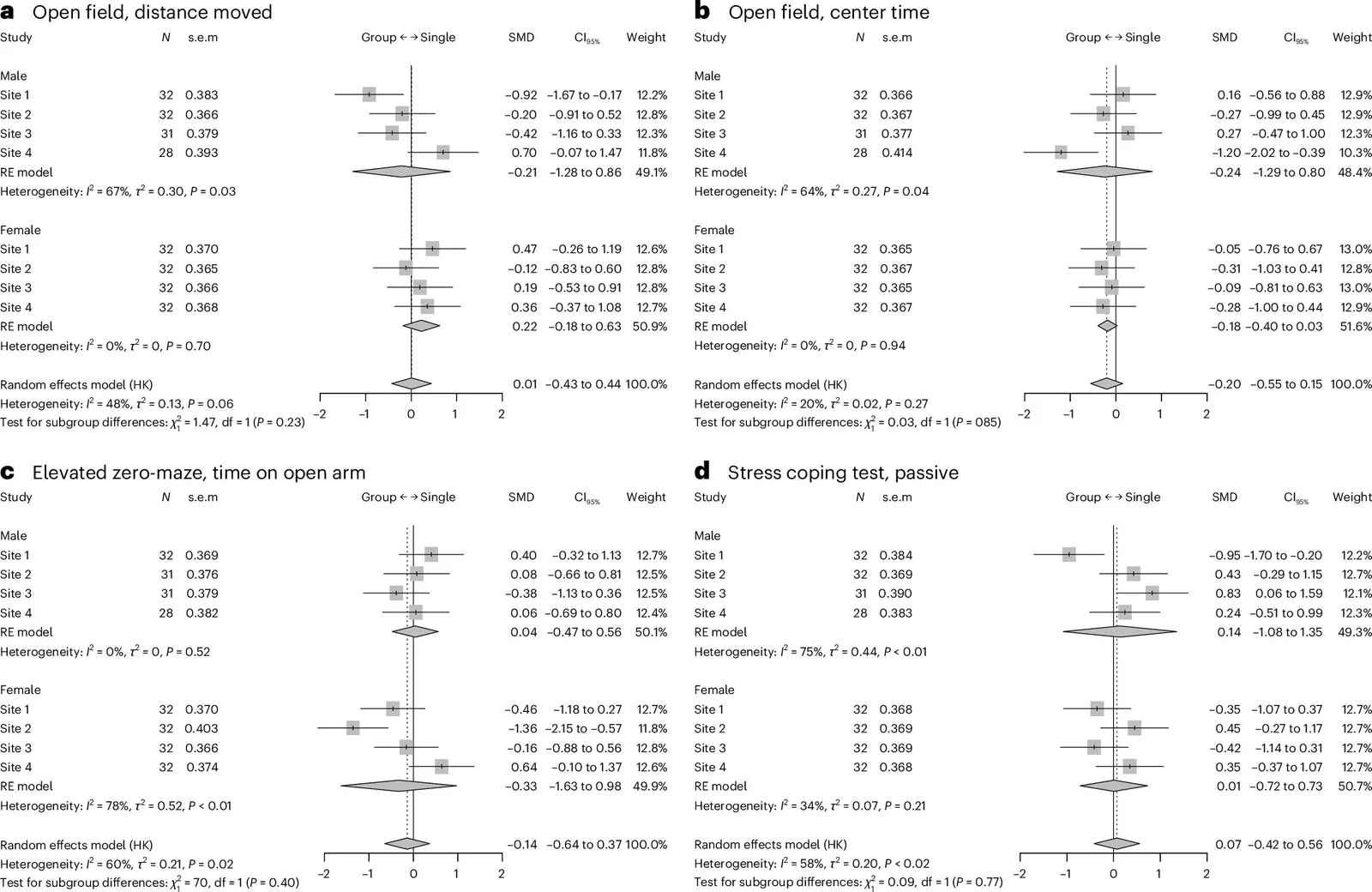
Severity-sensitive parameters in behavioral tests. Forest plots represent the effect size for each laboratory. Positive effect sizes indicate a predominant outcome in single-housed mice. Negative effect sizes indicate an outcome in favor of the group-housed mice. Significant results do not cross the original line. **a**, Distance moved in the open field test. **b**, Time spent in the center of the open field. **c**, Time spent on the open arms in the elevated zero-maze test. **d**, Time spent immobile floating as a passive coping in the forced swim test. Forest plot showing SMDs with CI_95%_ for each included study. Sample sizes (*N*) are shown for each study in the corresponding columns of the forest plot. The numbers of male/female animals (*n*) were as follows: single-housed site 1: 12/12, site 2: 12/12, site 3: 11/12, site 4: 12/12; group-housed site 1: 20/20, site 2: 20/20, site 3: 20/20, site 4: 16/20. Square sizes are proportional to the study weights, and diamonds represent the pooled effect estimates obtained using a random-effects model with the HK adjustment.

Locomotion and time spent in the center of the open field were not affected by social housing conditions (distance moved: *g* = 0.01, CI_95%_ −0.43 to 0.44, *t* = 0.05, *P* = 0.965; time in center: *g* = −0.20, CI_95%_ −0.55 to 0.15, *t* = −1.35, *P* = 0.219). For distance moved, the heterogeneity measure (*I*^2^ = 48%, *Q* = 13.37, df 7, *P* = 0.064) indicated that nearly half of the total variability in the effect sizes across studies could be attributed to between-study differences rather than random chance, suggesting moderate, although not formally significant, heterogeneity. No sex effects were detected (*Q* = 1.47, df 1, *P* = 0.23). We also detected no heterogeneity in the female subgroup related to the laboratory (*τ*^2^ = 0, *Q* = 1.43, df 3, *P* = 0.70). In the male subgroup, the data were substantially heterogeneous (*I*^2^ = 67%, *Q* = 9.12, df 3, *P* = 0.028). We found laboratory-specific alterations in male mice (*τ*^2^=0.30, *Q* = 9.12, df 3, *P* = 0.03). This was driven by the directionality in site 1, in which single-housed mice moved significantly less in the open field test (*g* = −0.92, CI_95%_ −1.67 to −0.17, *P* = 0.016), while in site 4, the group-housed mice moved less, although not significantly (*g* = 0.70, CI_95_% −1.28 to 0.86, *P* = 0.20). Hence, the factor laboratory induced stronger effects than the housing condition (Fig. 5a). Housing did not influence the time in the center (*g* = −0.20, CI_95%_ −0.55 to 0.15, *t* = −1.35, *P* = 0.219) nor did sex (*Q* = 0.03, df 1, *P* = 0.85). However, 20% of the variability (*I*^2^ = 20%, *Q* = 8.80, df 7, *P* = 0.27) could be attributed to between-study differences, although this heterogeneity was not statistically significant overall. Within the male subgroup (*I*^2^ = 64%, *τ*^2^=0.27, *Q* = 8.39, df 3, *P* = 0.04), the strongest deviation from the mean (*g* = −0.24, CI_95%_ −1.29 to 0.80) was the result from site 4, where male group-housed mice spent significantly more time in the center than group-housed mice (*g* = −1.20, CI_95%_ −2.02 to −0.39, *P* = 0.004). Site 4 was the only site where male group-housed mice spent more time in the center. In females, we did not detect heterogeneity (*I*^2^ = 0%, *Q* = 0.40), which indicated that differences did not have to be accounted for the different laboratories (Fig. 5b).

### Housing-induced substantial heterogeneity in anxiety-associated behavior

Time on the open arms in the elevated zero-maze data was particularly heterogeneous, with 60% of the variability originating from between-study differences (*I*^2^ = 59.7%, *Q* = 17.36, df 7, *P* = 0.015). Based on this, we could not detect an influence of social housing conditions on time spent on the open arms (*g* = −0.14, CI_95%_ −0.64 to 0.37, *t* = −0.65, *P* = 0.54). No differences between the sexes occurred (*Q* = 0.70, df 1, *P* = 0.4), but the heterogeneity between the sexes was striking. While the males displayed no heterogeneity (*I*^2^ = 0%), the attribution of variability to the different laboratories in females was 78%, despite the same set-up and standard operating procedure (SOP; *τ*^2^ = 0.52, *Q* = 13.49, df 3, *P* = 0.004). Particularly, the results from the site 2 laboratory (*g* = −1.36, CI_95%_ −2.15 to −0.57, *P* = 0.0007) and the site 1 laboratory (*g* = −0.46; CI_95%_ −1.18 to 0.27, *P* = 0.21) accounted for the heterogeneity. The opposite effect was detected in site 4, where single-housed females spent more time in the open arms (*g* = 0.64; CI_95%_ −0.10 to 1.37, *P* = 0.09) (Fig. 5c). Results for further parameters (number of exits on the open arm, crossings from one sheltered arm to the other and latency to first exit) were similar, with no significant differences due to sex, laboratory or housing (data not shown).

### Housing did not influence stress-coping behavior in the forced swim test in both sexes

The different laboratories had a substantial effect on stress-associated behavior (*Q* = 20.00, df 3, *P* = 0.0002); 58% of the total effect size could be attributed to the different laboratories (*I*^2^ = 58%, *Q* = 16.68, df 7, *P* = 0.02). The housing effect was insignificant (*g* = 0.07, CI_95%_ −0.42 to 0.56, *t* = 0.34, *P* = 0.74).

There were no sex-dependent effects (*Q* = 0.09, df 1, *P* = 0.77). However, within the sex subgroups, laboratory effects became apparent in male mice (*τ*^2^ = 0.44, *Q* = 11.87, df 3, *P* = 0.008). The data from site 1 were striking, with *g* = −0.95, CI_95%_ −1.70 to −0.20 compared with the overall male result of *g* = 0.14, CI_95%_ −1.08 to 1.35, *P* = 0.034. It was the only laboratory where male mice in single housing displayed more immobility than those in group housing. The results of male mice in site 3, however, showed a milder but opposite effect, *g* = 0.83, CI_95%_ 0.06 to 1.59, *P* = 0.034. The heterogeneity within the male subgroup was considerably high, with *I*^2^ = 75%. In females, no laboratory effects became apparent (*τ*^2^ = 0.07, *Q* = 4.56, df 3, *P* = 0.21), and heterogeneity was moderate with *I*^2^ = 34% (Fig. 5d).

### Individual and cage-based effects were a strong source of heterogeneity in body weight

The typical body weight difference between the sexes (*χ*^2^ = 100.2, *P* < 0.001) and over time (*χ*^2^ = 1,980.9, *P* < 0.001) was detected. Statistical analysis was performed using a linear mixed-effects model implemented with the lmer function in R. Significance was assessed using type III Wald *χ*^2^ tests from an analysis of deviance table.

When considering the cage and ID (nested) as a factor in our mixed model analysis, we found that the body weight of some animals deviated individually in specific cages. The nested factor cage/animal drove 49.5% of the total variance. However, the cage alone contributed 42.1% of the total variance. Therefore, a large part of the variance in the between-study data was attributed to the cage. By modeling this explicitly, the residual variance of the model could be reduced drastically (to 0.08%), allowing for more honest measurements of the uncertainty of the main effects. While there was substantial between-cage variability, the significant main effects remained strong enough in the presence of this variation.

**Fig. 6:**
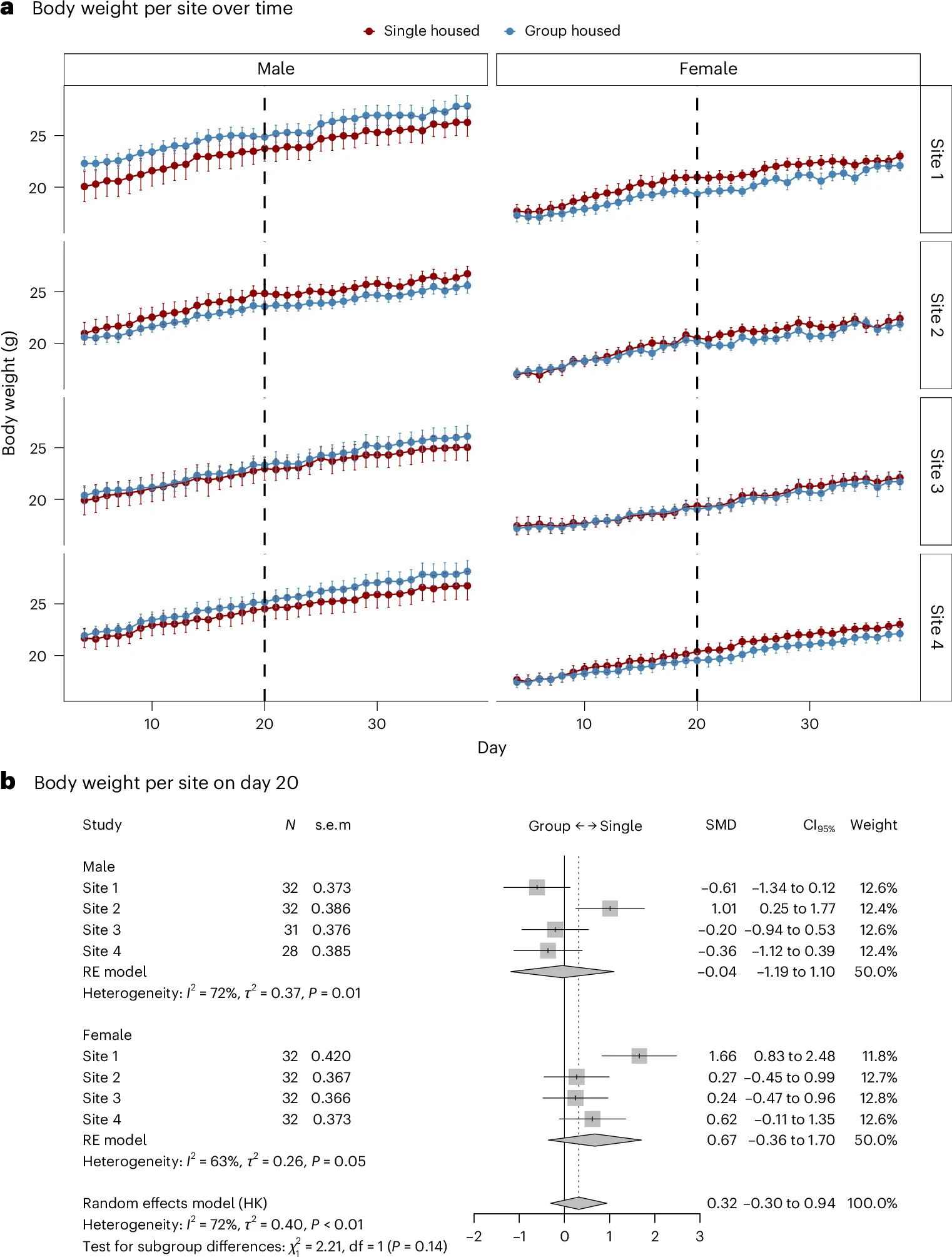
Body weight per site and sex during the study. **a**, The body weight of single (red) and group-housed mice (blue) in male (left) and female (right) mice. The body weight increases over time at all sites. The error bars represent the 95% confidence interval of the mean. **b**, On a representative day before experimental onset (day 20), no differences between the housing conditions were detected. Positive effect sizes indicate a predominant outcome in single-housed mice. Negative effect sizes indicate an outcome in favor of the group-housed mice. Significant results do not cross the original line. Forest plot showing SMDs with CI_95%_ for each included study. Sample sizes (*N*) are shown for each study in the corresponding columns of the forest plot. The numbers of male/female animals (*n*) were as follows: single-housed site 1: 12/12, site 2: 12/12, site 3: 11/12, site 4: 12/12; group-housed site 1: 20/20, site 2: 20/20, site 3: 20/20, site 4: 16/20. Square sizes are proportional to the study weights, and diamonds represent the pooled effect estimates obtained using a random-effects model with the HK adjustment.

Generally, the group-housed animals weighed 4.3 g less than the single-housed mice (*χ*^2^ = 406.48, df 1, *P* < 0.001; Fig. 6a).

In addition to our analysis over time, we performed an in-depth analysis on day 20 as a representative day before the experimental onset: the heterogeneity was again substantial (*I*^2^ = 72%; *Q* = 25.25, df 7, *P* < 0.001), and no housing-dependent effects were detected (*g* = 0.32, CI_95%_ −0.30 to 0.94, *t* = 1.21, *P* = 0.27).

However, the results from site 1 were striking: while male mice showed the smallest effect size between housing conditions (*g* = −0.61, CI_95%_ −1.34 to 0.12, *P* = 0.10), females showed the largest effect size (*g* = 1.66, CI_95%_ 0.83 to 2.48, *P* < 0.001). In site 2, group-housed male mice weighed significantly less than single-housed males (*g* = 1.01, CI_95%_ 0.25 to 1.77, *P* = 0.009). Body weight results at the other laboratory sites were not significant (Fig. 6b).

## Discussion

**Fig. 7:**
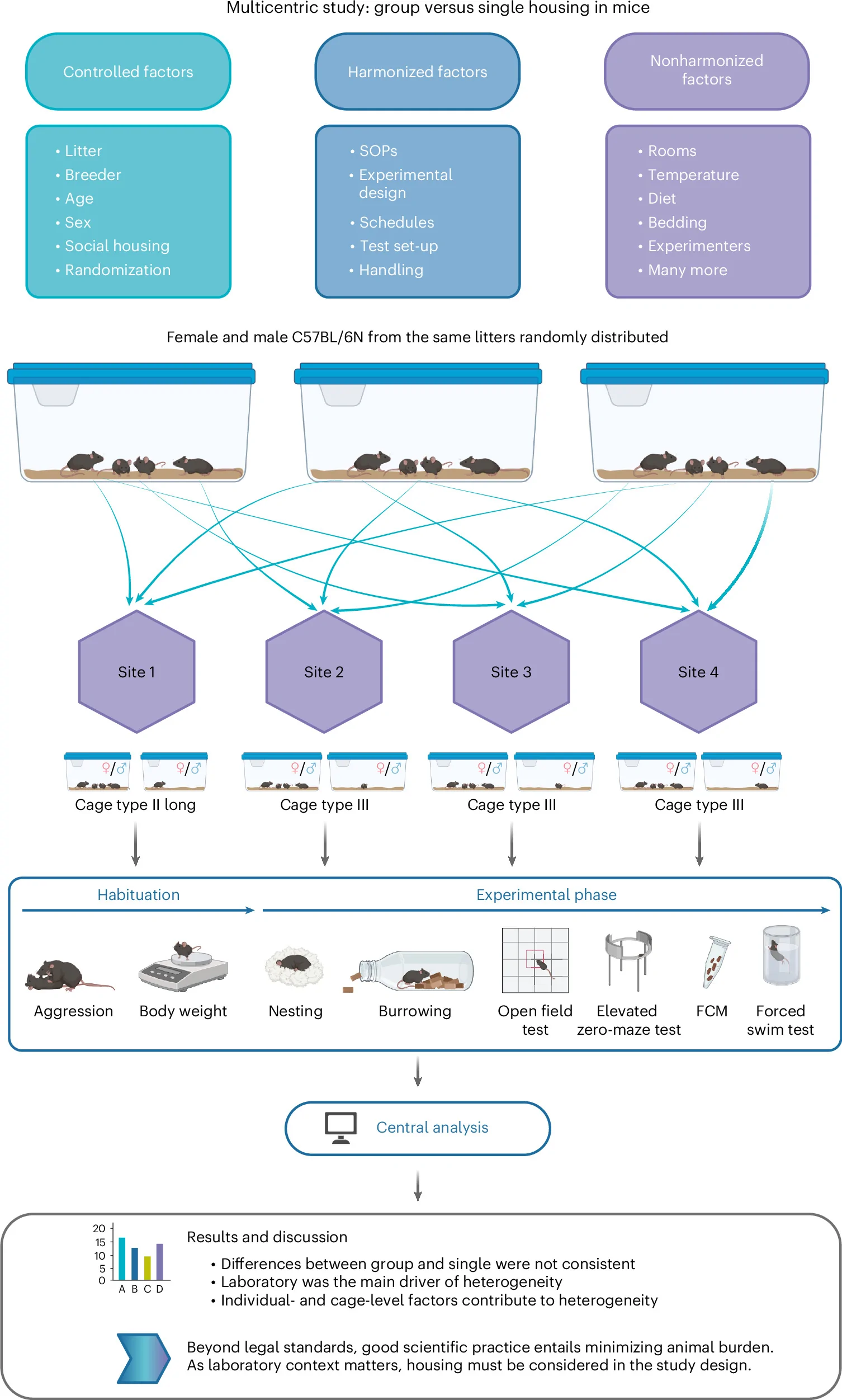
Summary of the multicentric study. In this multicenter study, we assessed the severity of single and group housing in male and female mice, which were housed in a sex-separated manner. For this, we observed physiological parameters (body weight and fecal corticosterone metabolite concentration) and behavioral readouts in the home cage (aggression, nest building and burrowing) and in behavioral set-ups (open field test, elevated zero-maze and forced swim test). We coordinated the origin of the animals, randomization and allocation to different housing forms (controlled factors). Home-cage-based parameters and behavioral tests were assessed using harmonized protocols, with data analysis conducted centrally (harmonized factors). Nonharmonized factors were laboratory based. In the interaction study of housing:sex:laboratory, the laboratory factor had the greatest impact. Figure created in BioRender; Mallien, A. (2026) https://biorender.com/c6svnek (2026).

Our study aimed to use a multicenter approach to investigate whether social housing substantially impacts the well-being of male and female laboratory mice compared with single housing. The mice were tested simultaneously and uniformly in four different laboratory sites. We expected robust effects across all locations, showing that social or individual housing would be the preferable choice for animal welfare, regardless of location. In addition, we expected female mice to show more substantial impairments caused by single housing. Figure 7 shows the study design, key findings and their relationships.

### Severity assessment

Outcome measures from behavioral analyses, biochemistry and physiology have proven to be the best approximation of the animal’s state^17^. Body weight, burrowing, nesting and FCM have been validated in previous research as sensitive indicators of overall well-being and stress-related changes. They provide insight into the animal’s state without invasive interventions, for example, performing nonessential behaviors^18^ or displaying stress-hormone release^19,20^. Alterations in these parameters can indicate decreased well-being.

These parameters have previously been shown to differentiate between varying levels of severity in mice when assessed using the Relative Severity Assessment (RELSA) score^21,22^. In the present study, we complemented them with measures to detect aggression and its consequences, potential alterations in locomotion, approach–avoidance conflict in anxiety-related behavior associated with height and coping behavior under stressful conditions. While cognitive assays (for example, judgment bias tasks and learning paradigms) would certainly provide an additional perspective on the welfare implications of housing conditions in mice, they were beyond the scope of the present study. Cognitive testing in mice requires extensive training and handling, which can introduce additional stressors and confound the interpretation of our primary behavioral and physiological outcomes. Nonetheless, there is evidence from mouse studies that social housing conditions can influence cognitive performance^10,23^. Thus, while cognition represents a highly valuable dimension for future work, in the current framework, we focused on behavioral and physiological indices that more directly addressed our research questions.

The behavioral and physiological parameters assessed showed no significant alterations due to housing. Among behavioral parameters, the only significant pooled effect was higher nest complexity in female mice under group housing; body weight also showed a significant main effect in the full longitudinal model, but this was highly site dependent rather than consistent. Apart from this observation, the expected trend toward impairment of the female mice due to individual housing did not occur. This suggests that female mice were similarly affected by single housing than males. However, male mice in the wild live solitary if they are not dominant or tolerated as subdominants in populations, while a solitary lifestyle of females is unusual^4^. Interestingly, this absence of pronounced social impairment in laboratory females may reflect domestication effects. In seminaturalistic studies, female wild mice form clear dominance hierarchies, whereas laboratory females (for example, C57BL/6) often fail to establish such hierarchies and show reduced aggressive and dominant behaviors. This suggests that the typical social organization observed in wild females has been largely attenuated in laboratory strains, probably as a byproduct of selection for docility and reduced stress responses, rather than as a deliberate elimination of aggression^24^. Another possible interpretation is that the severity associated with single housing of female mice may be overestimated under certain laboratory conditions, potentially influenced by anthropomorphic bias. However, it is important to note that neither single housing nor same-sex group housing reflects the natural social organization of mice, which typically involves interactions with both sexes and offspring. Therefore, both housing conditions should be considered compromises and may each contribute to reduced welfare depending on the context.

### Aggression

Aggression and the associated consequences have a decisive influence on the well-being of the mice. Kappel, Hawkins and Mendl^6^ published a comprehensive review of the harms and benefits of single and group housing for male mice and summarized recommendations for housing in groups. This review also displays a survey from the perspective of scientists and technicians directly involved in housing. It highlights the current discussion at any laboratory animal science meeting: the field is divided over whether single housing is a necessary evil to protect the mice from aggression or a source of detrimental social deprivation.

Our study confirms observations by scientists and technicians entrusted with animal care on aggression in adult male mice between different laboratory sites: while some detected nearly no attacks after cage change, it was pronounced in others. In site 1, attacks were scarce, but they were common in site 2. In site 4, the durations of attacks were short, but the consequences were so evident that we had to separate the mice. Female aggression was detected only at site 2. It was also the only site where increased whisker scores were reported in male and female mice. The whiskers of mice at site 2 were already impacted on arrival, leading to positive whisker scores even in single-housed mice. While the whiskers regrew over time, leading to improved scores in single-housed animals, the cumulative score continued to increase in group-housed males. In this study, we aimed to illustrate a real-life scenario, and barbering is a phenomenon that sometimes also occurs at breeders and hence on arrival. As barbering was declared a study outcome in our study, we included these animals in our study. We scored aggression directly after cage changes and at our daily routine during the light phase. In general, most attacks occur in the active, dark phase of the animals; therefore, most of the aggressive behavior may have been missed. The best solution to detect all the attacks would be an automated home cage assessment—a developing field with many promising approaches that will give us further insight into laboratory mice’s every day and social lives (for example, in refs. ^25–28^). However, it remains to be seen if automated home cage assessment is precise enough to score wounds similarly to the manual parting of the fur as done by an expert.

Certainly, aggression poses a risk to animal welfare that can lead to injuries or even death. In addition, pain and injuries change physiological parameters, influence data variability, reduce statistical power and external validity and raise the question of how standard mouse housing influences the validity of data in general^15,29^.

Many studies have aimed to determine relevant factors influencing aggression. In Table 1, we present the study’s set-up and compare it with many factors that have already been discussed in literature as influencing aggression and severity. Interestingly, many factors in those studies overlap. Still, the results are inconsistent: while a crowdsourcing approach of the NC3Rs^15^ found an indication with ear notches, this was not confirmed in a study by Theil et al.^14^. According to Theil et al.^14^, housing density was not listed as a significant influence, while Lidster et al.^15^ showed that a density of three mice per cage increased aggression-related incidents compared with other cage densities (odds ratio 5.17, *P* < 0.001). Factors relevant to aggression and group stability include the strain, the season of the year, bedding type, air conditioning system and parameters, environmental enrichment, positioning of racks and cleaning protocols^6,13,14,30^.

**Table 1:** Factors demonstrated to affect social housing of mice

Factor	Literature findings on interaction with housing	References	Present study
Strain	Genetic background is a key determinant of aggression. Some strains (for example, BALB/c, DBA/2 and CD-1/ICR) consistently display higher aggression. C57BL/6 and 129 are generally less aggressive	Weber et al.^34^	C57Bl/6NRj show a low rate of aggression and are a commonly used strain
	Clear strain effects, with some strains (for example, outbred CD-1/ICR and certain inbred lines) showing higher rates of aggression-related injuries than others (such as C57BL/6)	Lidster et al.^15^	
	Strain is the second strongest predictor of fighting after housing conditions	Thiel^14^	
Familiarity and kinship	Kinship: Sibling groups tend to exhibit more stable social relationships and, in some cases, less aggression—but this has not been confirmed in all studies	Weber et al.^34^ Lidster et al.^15^	Mice from the same litters were distributed across sites and randomly assigned to cages to minimize litter effects and reflect common procedures
	Male mice socialized before weaning show less aggression than those put together later. This effect was driven by familiarity, not genetic relatedness	Van Loo et al.^67^ Van Loo et al.^36^	
	Familiarity: nonsiblings raised together from an early age can form social groups just as well as siblings—familiarity seems to be more important than genetic relatedness	Kappel et al.^6^ Lidster et al.^15^	
Age	Males grouped before sexual maturity (approximately 4–6 weeks) show less aggression than those grouped later	Weber et al.^34^	We familiarized the groups before sexual maturity. They were 5 weeks old when assigned to groups (approximate sexual maturity of C57BL/6NRj mice)
	In CD-1 mice, early grouping before puberty resulted in more aggression, while other studies in other strains (for example, C57BL/6) often report that early grouping reduces aggression. This suggests a strain-dependent sensitivity to the timing of grouping	Bartolomucci^68^	
	Male mice are usually grouped together after weaning (approximately 3–4 weeks), with earlier grouping before sexual maturity being preferred to minimize aggression	Kappel et al.^6^	
Group size	Aggression increases in groups that are too large. Small groups of two to five male mice lead to more stable hierarchies and fewer injuries	Van Loo et al.^67^ Van Loo et al.^36^	Our mice were housed in groups of four or were housed individually
	Small-to-medium-sized groups (two to five animals) generally minimize aggression. Pairs can be socially understimulating but still exhibit low aggression. Groups of six or more increase the risk of injury and stress	Weber et al.^34^	
	Recommends three to five animals per cage, depending on cage size, strain and socialization. Group size interacts with kinship and age when grouping	Kappel et al.^6^	
	Laboratories typically use two to five male mice per standard cage. Groups of three have a higher prevalence of aggression than groups of four and five males	Lidster et al.^15^	
	Fewer aggression-related injuries in groups of three than two male CD1-mice	Jirkof et al.^52^	
Environment: cage size and enrichment	Larger cages can reduce aggression because there is more space for retreat and territorial niches. The effect is lost in very large groups, as the animals compete for resources	Van Loo et al.^67^ Van Loo et al.^36^	Site 1 could not house mice in type III cages like the others. Mice were housed in type II long. Cage sizes: recommended for groups of four Cages were equipped with aspen bedding and cotton nestlets
	Standard cage sizes (330–500 cm^2^) are sufficient for two to five male mice if the animals were socialized before puberty. Aggression increases when many animals are kept in a confined space or when there is a lack of places to retreat to	Kappel et al.^6^	
	Cage size interacts with group size and strain. For smaller groups, the effect of cage size is small; for larger groups, a larger cage can significantly reduce aggression	Weber et al.^34^	
	Higher risk for aggressive behavior of males housed in individually ventilated cages with corncob bedding. Also, there is a higher risk for aggressive behavior if cages are positioned in the upper rows of the cage rack or the rack is positioned parallel to a wall	Theil et al.^14^	
	Retreat areas (nests, platforms and tubes) are more important than absolute cage space; animals use them to reduce social tensions	Hurst and West^23^ Gaskill et al.^69^	
	Monopolizable resources promote aggression, while segmented enrichment (for example, multiple levels and nesting material) can reduce group conflicts	Kappel et al.^70^, Ratuski and Weary^71^	
	Nesting material instead of individual nest boxes to prevent monopolization and open structures or several identical hiding places prevent male aggression	Van Loo et al.^67^ Van Loo et al.^36^	
	Complex but monopolized enrichment increases aggression compared with socially equitable distributed environmental enrichment	Streiff et al.^72^	
	Running wheel-igloo increase aggression in CD-1 males	Howerton et al.^31^	
	Cage dividers can decrease aggression during hierarchy formation and lead to less despotic hierarchies and lower anxiety levels. Opaque dividers may increase aggression	Mertens et al.^45^ Tallent et al.^73^	
	Cage climbers are structural, group-compatible enrichment for large cages	Vogt et al.^30^	
	Nests compensate for cold and improve well-being in singly housed mice	Gaskill et al.^74^ Gaskill et al.^69^	
	Clustered enrichment can increase aggression in socially housed female mice	Akre et al.^75^	
Handling	Less stressful handling (tunnels and cupping) generally promotes calmer animals and improves experimenter–mouse interactions. This may indirectly support more stable group housing by reducing baseline stress and anxiety	Hurst and West^23^ Gouveia and Hurst^76,77^ Sensini et al.^78^	Although nonaversive methods improve welfare, we used tail handling to reflect common laboratory practice and maintain external validity
	Direct evidence for an effect on group dynamics is limited. In grouped male C57BL/6 mice, handling with tweezers/forceps increased aggression, whereas tail and tube handling were associated with lower levels of aggression	Mertens et al.^45^	
	More frequent, predictable handling led to fewer aggression-related injuries than weekly handling	Jirkof et al.^52^	
Season	Higher risk of aggression-related injuries in the summer months	Theil et al.^14^	The experiments were conducted from the end of September to November, avoiding the summer months
Cage-change protocol	Aggression decreases when nesting material is transferred and with spot cleaning rather than frequent full cage changes	Gray and Hurst^79^ Van Loo^35,80^	Cages were changed once per week. Bedding was transferred
	Transfer of bedding material can increase aggression; transferred nesting material and enrichment should be clean and free of urine to avoid promoting aggression	Van Loo^35^ Zidar et al.^80^	
	Cage changing is stressful; singly housed mice often show stronger stress reactions than group-housed mice	Lidster et al.^15^ Zidar et al.^80^	
Humidity and temperature	Stable environmental conditions improve well-being; stress-related conflicts in groups could be reduced	Meier et al.^81^	All sites follow the GV-SOLAS guidelines for housing
	Stress caused by extreme environmental conditions can promote aggression and social instability	Le Bras et al.^82^	
	Mice kept at standard laboratory temperatures (~20–24 °C) show increased stress marker levels (corticosterone), higher energy intake and altered behavior	Gaskill et al.^83^	
	Subthermoneutral temperatures promote stress, alter immune responses and may increase aggression in groups	Kokolus et al.^84^ Cannon and Nedergaard^85^	
	Group cuddling as a response to cold is an indication of social modulation through thermoregulation	Levy et al.^86^	
Food	Different feeding regimes (quantity, distribution and composition) influenced stress markers in subordinate mice and the level of aggression in groups of male mice	Bartolomucci et al.^68^	Sites 1 and 4 shared one feed and sites 2 and 3 shared another. Food was available ad libitum
	Engaging, structurally rich feed reduced aggression and stereotypical behavior, even in groups with dominant individuals	Kappel et al.^70^	
	Uneven distribution of feed/nesting material led to more fights; differences in feed composition can modulate resource availability and thus intensify group conflicts	Van Loo et al.^87^	
Sex of experimenter	Male experimenters induce stronger stress responses and stress-induced analgesia in mice, which may interact with housing-related stress. This makes experimenter sex a relevant environmental factor for group housing, particularly in male groups	Sorge et al.^88^ Bohlen et al.^89^	All experimenters were female
Ambient noise	Chronic noise interacts with housing; isolated mice are more vulnerable to corticosterone elevation	Turner et al.^90^ Rabat et al.^91^	No radios or white noise, but different background levels expected from corridors or neighboring rooms
	Noise and unpredictable sounds increase stress markers in rodents	Balcombe et al.^92^	
	Environmental noise can modulate behavior, activity and social interactions	Castelhano-Carlos and Baumans^93^	
Circadian rhythm, testing time and illumination	Social approach and agonistic behavior occur significantly more frequently and intensely in male mice during the dark phase than during the light phase	Arakawa et al.^94^	Experiments and handling: during the active dark phase. All sites followed the GV-SOLAS guidelines for housing. Illumination during experiments was harmonized
	Exploration and response to environmental enrichment are stronger in the dark phase, which is also relevant for the assessment of social behavior	Lewejohann^95^	
	Mice show more intense social interactions in dark phase but withdrawal and signs of stress in the light phase	Guzmán et al.^96^	
	Altered light intensity or light exposure during the dark phase disrupts circadian rhythms, elevates stress markers and modifies activity patterns in mice, which may in turn influence social interactions, aggression and group stability	Fonken et al.^97^ Bedrosian et al.^98^	

We housed mice in groups of four. Our experiments were performed from September to November, when aggression was reported to be least pronounced^14^. Resources such as food, water and enrichment such as rigid shelters and running wheels^31^ will probably be defended by the dominant male mouse, especially if they are not distributable^32–34^. To further reduce the potential of aggression, our mice therefore received only nesting material as an enrichment, easily distributable within the cage. However, nests are also resources that can be defended from other mice. Another factor potentially influencing aggression is identification-induced pain by ear clipping^33^. We avoided this by color-painting the tail of the mice for identification. We grouped the animals on arrival from the commercial breeder at the age of 5 weeks. For randomization, we did not leave the animals in their respective groups. We redistributed the mice from the same breeding facility to the four different laboratories and within the laboratories into the group or single housing conditions.

Furthermore, the weekly cage change can temporarily increase aggressive behavior, as fresh cages or cleaning eliminate established structures such as the nest site, latrines and scent marks^35–37^. Transferring nest material at cage change has been shown to reduce aggression in mice, while transferring soiled bedding, containing urine or feces, can increase aggression. As we wanted to score the nest complexity after cage change to assess the animals’ well-being, we chose to provide unused cotton nestlets according to Deacon^38^, and to transfer approx. 50 ml used litter during cage changes was an already established practice in our laboratories to reduce aggression.

Social housing is a critical matter for mice, yet the severity assessment that can be drawn from the outcome of behavioral and physiological parameters in this study was minor, and we did not observe any housing effects. Earlier studies have already painted a diverging picture. In 2024, Smolensky et al.^39^ showed that temporary single housing did not affect mice in a behavioral battery of tests, which assessed affective states and stress hormone release. However, minor effects on social behavior, cognition, thigmotaxis and body weight were detected in male and female mice. Other studies have shown increased corticosterone release^40,41^, but this was not reported by Smolensky et al.^39^ nor by Hunt and Hambly^42^. The FCM concentration was also inconclusive in our current multicenter approach. Body weight was reported to be altered by housing conditions in some publications^43–46^ but not in others^39,47^, and our findings similarly did not support a robust or generalizable housing effect: while the full longitudinal model detected a significant main effect of treatment (*P* < 0.001), this was accompanied by pronounced treatment-by-laboratory and treatment-by-sex-by-laboratory interactions, indicating that the direction and magnitude of the effect were highly site specific rather than uniform. Consistent with this, an independent day-20 snapshot analysis across sites found no significant pooled housing effect (*P* = 0.27), with only two of eight site-by-sex combinations (site 1 females, site 2 males) reaching individual significance.

Previous studies have reported anxiety-altering effects, most often detected by using elevated mazes, dark–light boxes and in the open field test. However, it is essential to mention that the time spent in the center of the open field does not necessarily correlate to anxiety unless validated for it^48^. Most studies have reported that social housing conditions affect anxiety-associated behavior in mice. These effects have been observed in either both or at least one sex with the predominant finding of an increase in anxiety^40,41,43,47,49–51^, but some also with a decrease^43,44^. In our study, housing did not induce significant effects but was associated with various results across sites, especially in female mice. Interestingly, most previous studies have not reported locomotion, a potential confounding factor for many other behavioral outcomes. Only Guo et al.^44^ have reported increased activity in males but not females due to social isolation, while Smolensky et al.^39^ reported no alterations in locomotion parameters. Social housing did not result in a general significant difference in locomotion in our multicenter study. Group-housed male mice in site 1 showed higher activity levels compared with the other groups; however, this effect was not observed in the pooled analysis of male studies, where the overall estimate did not differ significantly from zero, although laboratories differed subsantially in this subgroup (*P* = 0.03 for the laboratory subgroup effect within males). Interestingly, these animals displayed a similar pattern in the stress-coping parameter immobility, which was otherwise inconspicuous for this research. In several studies, housing influenced immobility time in the forced swim test, either decreasing it^44^ or increasing it^40,41,50^, and Smolensky et al.^39^ reported no visible alterations on immobility time through short-term solitary housing. Home-cage-based burrowing and nest complexity have been used to compare single and group housing before^46,52^. In our study, we did not observe a consistent overall effect of housing condition on nest complexity across all datasets. However, both the centralized photo-based analysis and the on-site assessments indicated a tendency toward higher nest complexity in group-housed cages, particularly in female mice and in some sites. The stronger effect observed in the blinded centralized analysis suggests that methodological differences in scoring may influence the detection of housing-related differences.

### Laboratory factors matter

In this study, we used a harmonized multicenter approach to determine the severity of social housing using current conventional housing standards in four laboratories. We wanted to investigate and confirm common observations, notably differences between laboratories in the number of attacks, the extent of aggressive behavior and the necessary separation of social groups. We also aimed to examine whether a more refined form of housing can be identified for each sex.

Given that social housing was thought to strongly influence the animals’ behavior, we expected the effects to be large enough to be detected under harmonized yet nonidentical circumstances. We expected different effect sizes but a general tendency toward detrimental effects of single housing in female mice and a more conflicted situation in male mice. However, the most persistent finding was the strong impact of the laboratory sites on the different results, despite the mice having the exact same origin and age, the harmonization of many factors, the use of identical laboratory equipment and SOPs. We do not know if introducing these measures led to better precision of the assessed parameters, as the overall laboratory-induced variability was very high. We intentionally did not adjust all factors in this study across the different laboratory sites to collect results that can echo real-world settings. The sites used different diets, bedding materials, environmental enrichment and cage sizes (Supplementary Table 1).

Many other factors remained undetermined and hence laboratory specific, for example, food and bedding, experimenters and background noise (Table 2). To display the usual differences between laboratories, further harmonization of conditions would have been counterproductive. This, of course, led to various potential risks and biases (Table 3). We expected that laboratory-specific effects, for example, diet choice, would influence body weight and make direct comparisons difficult. Diet can have different energy content and amounts of fiber (relevant for satiation), but also soy content, which can introduce phytoestrogens that may influence behavioral outcomes^53^, to name only a few factors. In this study, sites 1 and 4 used the same feed, as did sites 2 and 3. The feeds differed in phytoestrogen content (LasQdiet from Altromin is soy free, V1534-300 rat/mouse maintenance feed from ssniffis not; Supplementary Table 1), gross energy (15,9 MJ/kg versus 16,2MJ/kg) and fiber content (4,3 g/kg versus 4,9 g/kg). Our results do not indicate a direct comparability of outcomes due to feed, not even for the most obvious parameter, body weight. The sex of experimenters has been reported to be relevant for the outcome of behavioral analyses^54^. In our study, all experimenters were female, but of course, the teams in the different laboratories were different. While mice in three laboratory sites were housed in open-cage housing, the laboratory in site 1 additionally used filter tops owing to hygiene requirements in the facility. As this housing form was mandatory at the site, harmonization was not possible. The only alternative would have been to exclude the site from the analysis. Also, the bedding material in site 1 was excellent for creating nest sites, even without the cotton nestlets, which may have influenced the nest building drive and made the evaluation of the nests more challenging. This might explain the discrepancy between the live scoring and the scoring from photos. Differences such as this one might be a source of the frequent deviation of results in site 1. However, many other factors could have contributed (Table 1), including many that may still be unidentified. It is impossible to understand or attempt to standardize all husbandry parameters that might affect mice.

**Table 2:** Controlled, harmonized and nonharmonized factors in this study

Controlled factors	Harmonized factors between sites	Nonharmonized factors
Litter Breeder Age Sex Group or single housing Group size Randomization	Housing duration before tests Protocols/SOPs Test apparatus Tail handling Circadian rhythm 12/12 Time of test Transport in a home cage Cage change: 1× per week, transfer of old bedding	Humidity Temperature Rooms Food, diet and bedding Experimenters Transport to test rooms Cage size Illumination in the test room Background noise

**Table 3:** List of risks and potential biases in this study

Randomization	Animals from the same litters were shipped from the breeder to all sites. On arrival, we used randomization to allocate mice to cages and, hence, to the treatment group
Positioning in the room	Cages were randomly placed in the racks to avoid bias
Blinding of experimenters	Blinding was not possible, as housing conditions were visible. This is relevant because knowledge of the housing condition could unintentionally bias the observers’ recording or interpretation of the animals’ behavior
Blinding during analysis	Parameters were automatically assessed by software. For the central analysis, the expert was blinded (videos did not include cues). Data files included blinded numbers. Nest scoring was performed by nonblinded staff and the blinded expert
Information on incomplete outcome data	All dropouts were documented and reported. We had to exclude one group-housed cage in site 4 owing to aggression. The mice were separated once it was unreasonable to keep them in a group. The outcomes of these animals were excluded from the data analysis, except for the parameters related to aggression, wound, fur and whisker scores. As the observed aggression and its consequences were considered key study outcomes, these parameters were included in the analysis. One mouse in site 3 had to be euthanized owing to health reasons. No data on this mouse were included in this study
Experimental unit	The experimental unit of most parameters was the individual mouse. Only the evaluation of nesting, aggressive behavior and FCM was a cage-based comparison
Observer influence	Experimenters remained in the room during the open field, elevated zero-maze and forced swim tests but were behind a barrier and, therefore, not visible to the mice. Animals were observed via screen
Experimenters	All experimenters were female. Some of the experimenters were cat owners. Odors from pets, especially cats, are suspected to influence rat behavior. We strictly adhered to established sanitation procedures to prevent any contamination. All personnel wore dedicated laboratory coats or scrubs when handling animals, and rigorous personal hygiene is part of our routine
Expertise of experimenters	All experimenters had a background in behavioral biology or veterinary sciences. Most had at least 5 years of experience in behavioral assessment. Two experimenters had no previous experience with behavioral set-ups of the elevated zero-maze and the forced swim test but were always guided by an expert. Elevated zero-maze and forced swim tests were newly implemented at sites 1 and 4, and hence, experimenters were not previously trained in these specific tests. The colleagues were in daily contact with experienced experts from other sites during experimentation
Reporting	We report all assessed data. We comply with the FAIR principles (findable, accessible, interoperable and reusable)

To circumvent the problem of not being able to blind the experimenters to the housing conditions, the analysis was performed by a blinded expert using image-processing programs to analyze photos and video.

Of note, it is not only the laboratory that affects the data. We also noticed the unique behaviors of individual animals. Some mice buried bedding material into the burrowing bottles instead of emptying them from food pellets. Some mice did not pluck the nest material apart but pulled it entirely over themselves for protection and thus scored poorly in the nest evaluation. However, they did find a sensible but different solution. Furthermore, individual characteristics have a greater role in body weight than the social housing composition. Therefore, the impact of the individuality of the animals cannot be overlooked.

Inconsistent findings in outcome parameters across studies and even within this study indicate that the needs of the mouse may have a greater role than generally assumed, especially in an inbred line like C57BL/6. These individual differences in response to housing conditions suggest that a one-size-fits-all approach may not fully address the needs of every mouse. While group housing remains the general recommendation, in line with guidelines, for example from the GV-SOLAS or the Swedish 3Rs Center, housing decisions should be adapted on a case-by-case basis.

Valuable decisions can be made even before the project begins, in line with the recommendations of the Swedish 3Rs Center. These include several considerations: whenever possible, littermates should be housed together, with group formation ideally occurring before sexual maturity to promote stable social structures. Measures such as the targeted transfer of nesting material during cage changes can help reduce aggression and should be incorporated into routine husbandry. In studies requiring frequent cage changes, the risk of aggression increases, necessitating intensified monitoring or, if necessary, considering single housing. It is also important to minimize disturbances in the housing environment by limiting the number of handlers to reduce stress-induced aggression. In addition, choosing a less aggressive mouse strain, when possible, can further support animal welfare. These factors can be planned before the study and contribute to improving animal well-being as well as reducing experimental variability.

Harmonization and standardization have been challenged in science, and their problems and limitations have been brought forward multiple times^55–61^. Other options, such as heterogenization^62^, might often be better suited to generate robust and generalizable results owing to their high external validity. Of course, during the planning phase, we also considered whether a heterogeneous approach would be the best solution for this research question. However, we wanted to portray the environment and the most common laboratory situations, which are still standardized. And given that laboratories are a major influence^59,63,64^, we decided that mapping this ordinary/standardized approach was more representative on a greater scale. In fact, a general recommendation on housing based on a heterogenized approach might even be misleading and inappropriate for the respective laboratories.

A question that arises concerns the sensitivity of parameters. Were we unable to detect differences in severity because the chosen tests are not sensitive enough to measure possible smaller effects of the housing conditions? In this respect, a 2024 multicenter study questioned the nonharmonized use and the associated limitations of the Irwin score for characterizing drug-induced neurological side effects^16^. Similar to in our study, the results showed significant variability between laboratories, even though the experimental and environmental factors were also harmonized.

Such interlaboratory variability may arise from subtle, often uncontrolled differences in factors such as handler experience, micro-environmental parameters (for example, noise, vibrations and local room layouts) or differences in the interpretation and scoring of behavioral tests. These influences can affect animal behavioral and physiological outcomes in ways that cannot be fully mitigated by protocol harmonization.

We therefore assume that the tests were sensitive enough to detect severity, as previously shown. Nevertheless, the laboratory factor is so important that it overrides the smaller differences in responses in the animals, leading to higher or lower severity between the sites, especially with regard to aggressive behavior^65,66^. This finding also highlights a central aspect of reproducibility, which is the difficulty of obtaining comparable results across different laboratories, even when experimental protocols are harmonized. The pronounced laboratory effect observed here underscores that standardization alone does not eliminate site-specific influences. Rather than being purely a nuisance variable, such variability provides important information about the robustness and generalizability of severity assessment. Our multisite design thus not only quantifies the magnitude of laboratory-related variability but also emphasizes the need to interpret single-site findings with caution when extrapolating to other settings.

A limitation of our study is the use of a single mouse strain, C57BL/6NRj, which restricts the generalizability of the findings. While C57BL/6 is a widely used and well-characterized strain family, it is known that different strains—and even substrains of the C57BL/6-lineage—can exhibit subtle but meaningful differences in behavior and physiology. Including additional strains in future studies would strengthen the evidence base and provide a broader understanding of housing effects across genetic backgrounds.

### Why social housing still matters

In the absence of generalizable effects in this study, it remains unclear whether the housing has a substantial role for the mice. Kappel et al.^6^ emphasize that the choice of housing must be highly context dependent, “including strain, age, social position, life experiences, and housing and husbandry protocols. It is important to recognize this and evaluate what is preferable from animal welfare and ethical perspectives in each case.” Does a lack of changes in tests for affective states mean the mice are in a ‘normal’ affective state? First of all, snapshot analysis such as elevated maze testing can give us an idea of the anxiety trait of the mice, but it will not be as meaningful as long-term severity assessment in an automated home cage^25^. Also, even if the affective state is unaltered and there are no indications of depression-associated alterations^39^, other parameters support the notion that social housing does influence the animals, such as body weight and sociability.

Our results do not provide evidence against group housing of female mice under the conditions tested; however, this should not be interpreted as evidence that this housing condition is optimal. It is more complex for male mice because of the possible laboratory-specific rate of aggression among cage inhabitants. Male mice have been shown to prefer social contact over empty cages^67^ but prefer nesting material over social contact^11^. In addition, in a severity-related cognitive task of reward assessment, male mice in group housing displayed similar deficits to males chronically treated with corticosterone^10^. At the same time, male mice housed in individual cages in an open and conventional set-up performed better, indicating that they are less negatively impacted^10^. Of course, this is only one observation, but it challenges the general opinion. More studies are needed to confirm this observation, particularly given our finding that the laboratory itself is a key influencing factor.

It is noteworthy that stable dominance hierarchies develop in domesticated laboratory males but not in females, whereas both sexes establish stable hierarchical structures in wild mice^24^. Also, in the same study, the personalities and archetypes of wild mice in the hierarchy are described. This cannot be observed in the current standard housing of laboratories and would require more natural enclosures^24^. The importance of enclosures and cages that allow more natural behavior is becoming increasingly evident; not only as a measure of refinement but also to improve scientific validity or the possibility of new observations and analyses. While seminatural enclosures or colony cages have not yet become the standard, more and better enrichment is provided nowadays. Whether or not male and female mice should be housed under identical standard conditions for comparability within a study should be decided on the basis of the specific circumstances of the laboratory. This means that females may be more affected by social deprivation than males and that male mice may experience harm owing to the social stress of dominance–subordination, resulting in physiological and behavioral consequences. Alternatively, male mice may experience reduced negative affect under certain laboratory conditions when housed individually^10^. Although our data suggest no difference in severity between the sexes, they were profoundly confounded by the laboratory effect.

Importantly, when interpreting these findings, it must be considered that both single housing and same-sex group housing in standard laboratory cages represent artificial conditions that deviate substantially from the natural social structure of mice. In the wild, male and female mice typically interact within mixed-sex groups, including offspring. As such, it remains unclear which laboratory housing condition more closely reflects the animals’ needs, and it is plausible that both conditions may impose different, but relevant, welfare burdens. This perspective should be considered when drawing conclusions about the relative severity of housing conditions.

## Outlook

The laboratory site contributed the most to the severity outcome in single and group housing. We suggest that the best housing type for each laboratory can only be found by and within the institution under the current husbandry conditions. While guidelines emphasize group housing of mice as a general recommendation, they also recognize exceptions where single housing may be necessary. In practice, however, group housing is sometimes treated as a default requirement, with single housing regarded as a burden that requires justification. This practice is not fully supported by evidence, as our data and recent studies^10^ indicate that housing preferences and welfare outcomes depend strongly on laboratory context. The current guidelines recognize that although mice should be kept in harmonious groups, it is not always possible. However, this does not relieve scientific leads, experimenters, animal facility heads and animal care staff from their responsibility to determine the best possible housing conditions for their animals and to repeatedly assess them. New findings on social husbandry should always be considered to further refine husbandry. A critical examination of the topic is an obligation for laboratories if they want to characterize the main effects of housing on the animals. Further research is necessary, and as the evidence base continues to grow, systematic meta-analytic approaches may help uncover broader, robust effects that are not immediately apparent.

## Methods

### Experimental design

We compared severity-associated changes in behavior and stress hormone release in group- and single-housed mice of both sexes in four different laboratory sites. The design considered controlled, harmonized and nonharmonized factors across research sites (Table 1).

After 25 days of initial habituation to the new housing conditions, we simultaneously assessed severity-associated parameters in all laboratories (Fig. 1).

### Animals and housing

Each laboratory received 32 male and 32 female C57Bl/6NRj mice at 5 weeks of age from Janvier Labs in a coordinated delivery: mice from the same litters were distributed to different locations. At each site, the animals were randomly allocated to single or group housing on arrival and remained in that condition throughout the project. Groups consisted of four individuals. Cages were randomly distributed in the racks in the housing rooms to avoid housing all group- or single-housed animals in one area or on one level. For all mice, acoustic, olfactory and visual contact to neighboring cages was possible. Likewise, the order of animals for behavioral tests outside the home cage was randomized each time (simple randomization; R version 4.1.156^99^ or randomizer.org). Blinding of experimenters was not possible owing to housing-based evaluation (Table 3). All animals had ad libitum access to food (Supplementary Table 1) and tap water. Each cage contained bedding material and cotton nestlets (Supplementary Table 1)—one in single-housed cages and two in group housing. See Supplementary Table 1, which lists nonharmonized factors, for further details on housing conditions. We aimed to harmonize cage sizes across all participating laboratories; however, this was not feasible owing to logistical and regulatory constraints. One site had no type II cages available, another faced a shortage of type III cages and at the third site, a change in regulatory approval would have been required. As this process is typically slow and substantial time had already been invested in preparation, we decided not to delay the project further and chose to integrate ‘cage size’ as a nonharmonized factor. Aggression and body weight were closely monitored during cage changes and included in the daily inspection routine within the first 2 h after the light change. The mice were habituated to the housing conditions for 25 days before the onset of behavioral experiments (Fig. 1). Although more gentle forms of mouse handling are available, we chose the currently most common form, tail handling, for comparability with the usual conditions in most laboratories^100^. The animals were housed on a light–dark cycle, which differed in times between the sites. However, all interventions were performed on the same schedule adjusted to the laboratory-specific light cycle times: handling, body weight assessment and cage changes within the first 2 h after light change; burrowing and nesting in the last 2 h before the light change to the dark phase; and behavioral tests outside the home cage started 2 h after light change to the light phase. None of the facilities involved in this project featured dimming phases to support the circadian rhythm. All experimenters were female.

The experiments were approved by the respective local authorities according to the German Animal Welfare Protection Law (LANUV A: AZ81-02.04.2020.A377; Regierungspräsidium Karlsruhe HD: 35-9185-81-G69-20 and MA 35-9185-81-G81-19; government of Upper Bavaria MÜ: ROB-55.2-2532.Vet_02-19-31). The testing sites were alphabetized as CIMH Mannheim, IBF Heidelberg, LMU Munich, and RWTH Aachen (Institute for Experimental Molecular Imaging). We adhered to the 3Rs throughout study design and execution. In addition, we considered the principles of the Basel Declaration^101^ and followed the Animal Research: Reporting of in Vivo Experiments (ARRIVE) 2.0 guidelines^102^ in reporting.

### Physiological parameters

#### Body weight

Body weight was assessed at arrival and starting on day 6 every weekday, always within 1–2 h after light change. The mouse was lifted by the tail and placed in a container on a scale to measure body weight.

#### Fecal corticosterone metabolites

For fecal corticosterone metabolite concentrations, samples were collected for 24 h in the home cages directly on days 43 and 50. The feces were frozen at −20 °C and transported to the Central Institute of Mental Health, Mannheim, Germany, where the extraction was performed^103^. The final measurement was performed in the laboratory in Vienna, Austria^19,78^ as previously described. In brief, homogenized and dried feces were extracted with 80% methanol. The samples were analyzed using a well-established and validated 5α-pregnane-3β,11β,21-triol-20-one enzyme immunoassay. While the corticosterone metabolites concentrations on day 43 may have been additionally influenced by the forced swim test conducted 19 h previously, we expected the measurements on day 50 to reflect the effect of housing conditions solely. As we found no differences in corticosterone metabolite concentrations between the 2 days, we merged the data for analysis for each experimental unit.

### Behavioral tests

Information on light intensity for behavioral analysis is presented in Supplementary Table 2. In experiments outside the home cage, the mice were habituated to the testing room for 30 min, except for the forced swim test. These tests started 2 h after the lights came on across all sites. The apparatus was cleaned thoroughly with 70% ethanol between subjects. Videos of the open field, elevated zero-maze and the forced swim test were centrally analyzed by an expert experimenter (more than 15 years of experience in the analysis of behavioral data) from the Central Institute of Mental Health in Mannheim for all laboratories using the image-processing program EthoVision XT 15 (Noldus Information Technology). During the test, the experimenters remained in the experimental room or an attached anteroom and watched the behavior indirectly via video recordings. The mice could not see the experimenters.

#### Burrowing

A total of 2 h (±1 h) before the dark phase, the mice were placed in individual burrowing cages (same floor size as home cages, identical and clean bedding material, access to water but no food in the food hopper) for 2 h daily over five consecutive days. The burrowing cages were not cleaned between trials to keep the individual’s scent marks, but burrowed pellets were removed after the daily test. Each cage was allocated to an individual mouse. The cages were randomly positioned during the testing phase to guarantee that not all mice from group-housed conditions were in one area and that those from single-housed conditions were in another. The burrowing cage included burrowing bottles (20 cm long, entrance 3.5 cm in diameter, and 2 cm above the floor) filled with 200 g of their familiar food pellets. After 2 h, the weight of the remaining pellets inside the bottles was used to evaluate burrowing performance, and mice were transferred back to their home cage^22,103,104^.

#### Open field test

The mice were placed in an unfamiliar white plastic arena (50 cm × 50 cm), illuminated from above for 10 min. Four mice were tested simultaneously in separate chambers without visual contact. The activity was recorded from above with a camera. Assessed parameters included the distance moved and the time spent in the center (defined as >10-cm away from the walls)^53,105^.

#### Forced swim test

The mice were transferred to a glass cylinder (diameter 13 cm, height 23 cm) filled with 24 °C tempered water to a height of 12 cm for 6 min (ref. ^53^). The animals were acclimatized to the testing room for 20 min. Four mice were tested simultaneously in separate chambers without visual contact. This test was conducted twice, with a 24-h intertrial interval. The water was changed after each animal. Under close observation, the animals were placed in front of infrared warming lights for 15–20 min after each trial, to ameliorate hypothermia. The behavior was video recorded from the side. Latency to immobility and total time spent immobile were measured.

#### Elevated zero-maze test

The mice were introduced into one of two opposing walled compartments (wall height 10 cm) of a zero-shaped gray plastic runway (outer diameter 46 cm, width 6 cm, height 50 cm above the floor) laminated with grip tape without edging to prevent the mice from slipping. The mice did not climb on the walls nor did any of them fall off the maze. The mice´s exploratory activity was recorded for 5 min from above. The experienced experimenter manually evaluated the latency to the first exit, the number of exits and the time spent on open arms for all laboratories’^53^ recordings. Manual scoring was required, as the contrast between the arena and the mice was insufficient for detection by the tracking system.

#### Wound, fur and whisker score

The experimenters evaluated the condition of the mice concerning wounds, fur and whiskers directly before the cage change once a week. For the wound score, the fur was examined by parting it with a cotton swab to get a better view of possible wounds. The wound score was determined as score 0 = none, score 1 = rough area(s), score 2 = superficial wound or <1 cm length/diameter and ≤3 wounds, score 3 = deep wound or 1–1.5 cm length/diameter and ≤3 wounds, and score 4 = ulceration or >3 wounds or >1.5 cm length/diameter. The fur and whiskers were scored separately on the basis of hair/vibrissae loss: score 0 = 0%, score 0.5 ≥30%, score 1 <50%, score 1.5 <75%, or score 2 = 100%. The cumulative score is the sum of scores on the respective testing day (fur and whisker score) or week (wound score).

#### Aggression

Aggression was assessed after cage changes. The animals were transferred with their familiar cage mates into the fresh cage and observed for 10 min. We documented the latency and duration of the first attack as well as the total number of attacks. Observed attacks included running aggressively toward conspecifics, making physical contact to push, bite or mount them. Mounting between same-sex cage mates in this context was interpreted as a form of dominance behavior rather than sexual motivation. When there was an interval of 1–2 s between attacks, they were counted as individual attacks, and the total number of attacks was scored. Tails were recently marked, making it easy to distinguish the mice. It was equally possible to follow all fights within one cage simultaneously, as there was mostly only one aggressor. Cages were evaluated sequentially, with only one cage being assessed at any given time.

#### Nesting

Nest building was assessed in the home cage on the day of cage changes. The mice were transferred to a clean new cage with one (single housing) or two (group housing) cotton nestlets on the left back of cage 2 h (±1 h) before the dark phase. The nest was scored at the end of the dark phase (±1 h). Scoring was modified from Deacon^38^ and Jirkof et al.^106^ (Supplementary Table 3). We compared on-site scoring with the expert´s scoring of unassignable photos (see Fig. 4c,d for results).

### Euthanasia

At the end of the experiment, a blood sample was collected via heart puncture under deep isoflurane anesthesia. Immediately after blood collection, mice were euthanized by cervical dislocation in deep isoflurane anesthesia.

### Statistical analyses

The experimental unit was the individual animal for all individually assessed parameters (burrowing, open field, elevated zero-maze, forced swim test, body weight, wound score, fur score and whisker score). For cage-based behaviors or physiological parameters, the experimental unit was the cage (nesting, fecal corticosterone metabolites and aggression). We excluded one mouse owing to health problems on site 3. The separation of one group was urgently necessary owing to aggression on site 4 at day 21. After separation, the animals no longer shared comparable housing conditions, and their data were therefore largely excluded from the analyses. Only parameters considered critical for the study outcome up to cage separation, such as aggression, wound score, fur score and whisker score, were included.

The programs G*Power^107^ from the University of Düsseldorf and the free statistical software R were used to calculate animal numbers. The calculation was based on the estimated effect sizes of all assessed parameters. Therefore, parameters with repeated measures (for example, burrowing and body weight) required a ‘repeated measures between factors’ design to statistically differentiate between the four groups. In addition, within–between interactions and within factors were analyzed whenever appropriate. The ‘Cohen 1988’ setting (80% power and *α* = 5%) was used to calculate the effect size, not the usual setting in G*Power. The parameter with the largest sample size was body weight (*f* = 0.4) in the within–between interaction with *n* = 112. To determine the sample size for a multicenter study, the intracluster correlation coefficient was used. Therefore, a realistic intracluster correlation coefficient of *ρ* = 0.09 was assumed to calculate the sample size for the laboratories. Each laboratory effectively required *n* = 61.15 ≈ 62 animals. The experiments were to be conducted in group housing with four animals per cage, resulting in an adequate sample size of *n* = 64 per laboratory site. For cage-based assessment, each site was planned with five groups and 12 single home cages per sex (Supplementary Table 4).

The analyses were performed in the R software (v4.3.1)^108^, and the following packages were used in the study: metafor^109^, dplyr^110,111^, multcomp^111^ and ggplot2^112^.

The multicentric data were pooled using the escalc function from the metafor package^113^. Parameters with repeated measures, for example, burrowing performance, fecal corticosterone metabolite concentration or nest scores in the nest test, were pooled for analysis. This was performed independently for each laboratory. The effect sizes were calculated as standardized mean differences (SMD) with the Hedges *g* correction and the sampling variances. Effect sizes between housing conditions were calculated directly in cases with only one measurement day.

An inverse variance meta-analysis was performed, with the laboratory, housing and sex parameters as subgroups for each quantitative outcome measure. To estimate the between-study variance (*τ*^2^), the restricted maximum likelihood method was used. The confidence intervals were calculated using the *t*-method, assuming a *t*-distribution. Consequently, owing to small sample sizes, the Knapp–Hartung method was used to adjust the test statistics and the CI_95%_. The meta-analysis results for each subgroup parameter were displayed as forest plots, indicating the within and between groups’ heterogeneity information and the individual and overall random effects with CI_95%_.

In addition, we analyzed the data with a multilevel linear mixed-effects model to estimate within- and between-subgroup effects regarding the multilevel structure of the study. The model used a diagonal covariance structure for the pooled effect sizes. The subgroup parameter defined the inner structure of the random effects, and the laboratory and the study ID defined the outer structures. Again, test statistics and CI_95%_ were determined with two-sided *t*-tests. For post hoc analyses, general linear hypotheses were generated with a contrast matrix. The family-wise error rates for multiple pairwise comparisons were adjusted with the Tukey procedure.

### Reporting summary

Further information on research design is available in the Nature Portfolio Reporting Summary linked to this article.

## Online content

Any methods, additional references, Nature Portfolio reporting summaries, source data, extended data, supplementary information, acknowledgements, peer review information; details of author contributions and competing interests; and statements of data and code availability are available at https://doi.org/10.1038/s41684-026-01805-7.

## Supplementary information

Supplementary Information Supplementary Tables 1–4.

Reporting Summary

## Data Availability

All raw datasets of the study are available via figshare at https://doi.org/10.6084/m9.figshare.31861540 (ref. ^114^).
