# Supplementary material for: Laboratory conditions matter: social housing in male and female laboratory mice in a multicenter study approach
Source: Lab Anim (NY). 2026 Oct 13;55(11):441–57. doi: 10.1038/s41684-026-01805-7 (PMC13710406; doi:10.1038/s41684-026-01805-7)
Supplement: Supplementary file 1 [file 41684_2026_1805_MOESM1_ESM.pdf]

lab animal

Supplementary information

https://doi.org/10.1038/s41684-026-01805-7

Laboratory conditions matter: social housing in male and female laboratory mice in a multicenter study approach

In the format provided by the authors and unedited

Supplementary Information

Supplementary Table 1

Non-harmonized factors.

	Site 1	Site 2	Site 3	Site 4
**Food**	V1534-300 Rat/mouse maintenance 10, Ssniff Spezialdiäten GmbH, Soest, Germany	LASQCdiet Rod16, Altromin Spezialfutter GmbH & Co. KG, Lage, Germany	LASQCdiet Rod16, Altromin Spezialfutter GmbH & Co. KG, Lage, Germany	V1534-300 Rat/mouse maintenance 10, Ssniff Spezialdiäten GmbH, Soest, Germany
**Bedding**	LIGNOCEL Wood Fibers, J. Rettenmeier & Söhne GmbH+Co KG, Rosenberg, Germany	ABEDD LT-E-001, ABEDD, Vienna, Austria	Abedd Espen MIDI, ABEDD, Vienna, Austria	Safe Bedding, Premium Scientific Bedding, J. Rettenmaier & Söhne GmbH + Co KG, Rosenberg
**Cage type**	type II long (floor area: 530 cm^2)	type III (floor area: 800 cm^2)	type III (floor area: 800 cm^2)	type III (floor area: 800 cm^2)
**Housing condition**	filter tops	conventional	conventional	conventional
**Nesting material**	Nestlets, cotton fiber, 5 x 5 cm squares, Zoonlab GmbH, Castrop-Rauxel, Germany	Nestlets, cotton fiber, 5 x 5 cm squares, Zoonlab GmbH, Castrop-Rauxel, Germany	Nestlets, cotton fiber, 5 x 5 cm squares, Zoonlab GmbH, Castrop-Rauxel, Germany	Nestlets, cotton fiber, 5 x 5 cm squares, Zoonlab GmbH, Castrop-Rauxel, Germany
**Cage preparation**	Nestlets, food and cages autoclaved	Nestlets, food and cages autoclaved, water filtered and irradiated	Food and cages autoclaved	Acidic cage cleaning (60 °C)
**Light-dark rhythm**	Summer 07:00–19:00; winter 07:00–19:00	Summer 06:00–18:00; winter 05:00–17:00 (switch on day 33)	Summer 06:00–18:00; winter 05:00–17:00 (switch on day 33)	Summer 06:00–18:00; winter 05:00–17:00 (switch on day 33)
**Sex-specific room**	no	no	yes	yes
**Temperature & humidity**	22 ± 2 °C, temperature- and humidity-controlled air condition, located in basement	One outer wall (additional heat), female rack standing in front of it	22 ± 2 °C, 55 ± 10%, controlled air condition, located on first floor	22 ± 2 °C, 55 ± 10%
**Other mice**	Mice from other studies in IVC in the same room	Only animals of this study in housing rooms and sentinels	Only animals of this study in housing rooms	Females: mice from another study and sentinels in the same room, all females; Males: no further animals
**Experimental room**	Behavior analysis in directly connected space	Behavioral analysis directly next door, not directly connected	Behavioral analysis directly next door, not directly connected	Behavioral analysis directly next door, not directly connected
**Additional information**	—	—	—	16 female mice arrived 2 days later than the rest

Supplementary Table 2

Illumination in behavioral analysis.

Site	Open field illumination	O-maze illumination
Site 1	25 lux from above	25 lux from above
Site 2	25 lux from above	25 lux from above
Site 3	25 lux from above	25 lux from above
Site 4	25 lux from above	25 lux from above

Supplementary Table 3

Nest score, modified from Deacon (2006) and Jirkof et al. (2013).

Score	Description
Score 1	Nestlet is almost untouched (>90% intact).
Score 2	Nestlet partly torn (50–90% intact).
Score 3	Nestlet mostly torn (50–90% torn):<br>• <50% intact<br>• >90% of the nestlet is in one quadrant of the cage.<br>• The cotton is scattered and not formed into a nest.
Score 4	Nest is identifiable but flat (>90% torn):<br>• The cotton is within one quadrant of the cage.<br>• The nest is flat: <50% of the rim is higher than the height of a sleeping mouse.
Score 5	Almost perfect nest (>90% torn). Nest resembles a bowl: >50% of the rim is higher than the height of a sleeping mouse.
Score 6	Perfect nest.

Deacon, R. Assessing nest building in mice. *Nat Protoc* 1, 1117–1119 (2006). doi: 10.1038/nprot.2006.170

Jirkof P, Fleischmann T, Cesarovic N, Rettich A, Vogel J, Arras M. Assessment of postsurgical distress and pain in laboratory mice by nest complexity scoring. *Laboratory Animals*. 2013;47(3):153-161. doi:10.1177/0023677213475603

Supplementary Table 4

Sample sizes used for analysis.

	Total number of included mice (male/female)	Experimental units, individual (male/female)	Experimental units, cage-based (male/female)
**Single housed**			
Site 1	12/12	12/12	12/12
Site 2	12/12	12/12	12/12
Site 3	11/12	11/12	11/12
Site 4	12/12	12/12	12/12
**Group housed**			
Site 1	20/20	20/20	5/5
Site 2	20/20	20/20	5/5
Site 3	20/20	20/20	5/5
Site 4	16/20	16/20	4/5
