# Supplementary material for: Laboratory conditions matter: social housing in male and female laboratory mice in a multicenter study approach
Source: Lab Anim (NY). 2026 Oct 13;55(11):441–57. doi: 10.1038/s41684-026-01805-7 (PMC13710406; doi:10.1038/s41684-026-01805-7)
Supplement: Supplementary file 2 [file 41684_2026_1805_MOESM2_ESM.pdf]

Corresponding author(s): Anne Mallien

Last updated by author(s): Jul 30, 2026

Reporting Summary

Nature Portfolio wishes to improve the reproducibility of the work that we publish. This form provides structure for consistency and transparency in reporting. For further information on Nature Portfolio policies, see our Editorial Policies and the Editorial Policy Checklist.

Statistics

For all statistical analyses, confirm that the following items are present in the figure legend, table legend, main text, or Methods section.

n/a	Confirmed	
☐	☒	The exact sample size (*n*) for each experimental group/condition, given as a discrete number and unit of measurement
☐	☒	A statement on whether measurements were taken from distinct samples or whether the same sample was measured repeatedly
☐	☒	The statistical test(s) used AND whether they are one- or two-sided<br>*Only common tests should be described solely by name; describe more complex techniques in the Methods section.*
☐	☒	A description of all covariates tested
☐	☒	A description of any assumptions or corrections, such as tests of normality and adjustment for multiple comparisons
☐	☒	A full description of the statistical parameters including central tendency (e.g. means) or other basic estimates (e.g. regression coefficient) AND variation (e.g. standard deviation) or associated estimates of uncertainty (e.g. confidence intervals)
☐	☒	For null hypothesis testing, the test statistic (e.g. *F*, *t*, *r*) with confidence intervals, effect sizes, degrees of freedom and *P* value noted<br>*Give P values as exact values whenever suitable.*
☒	☐	For Bayesian analysis, information on the choice of priors and Markov chain Monte Carlo settings
☐	☒	For hierarchical and complex designs, identification of the appropriate level for tests and full reporting of outcomes
☐	☒	Estimates of effect sizes (e.g. Cohen's *d*, Pearson's *r*), indicating how they were calculated

Our web collection on statistics for biologists contains articles on many of the points above.

Software and code

Policy information about availability of computer code

Data collection	EthoVision XT 15 (Noldus Information Technology, Wageningen, the Netherlands
Data analysis	G*Power<br>R software (v4.3.1) and R packages: metafor, dplyr, multcomp, ggplot2

For manuscripts utilizing custom algorithms or software that are central to the research but not yet described in published literature, software must be made available to editors and reviewers. We strongly encourage code deposition in a community repository (e.g. GitHub). See the Nature Portfolio guidelines for submitting code & software for further information.

Data

Policy information about availability of data

All manuscripts must include a data availability statement. This statement should provide the following information, where applicable:

- Accession codes, unique identifiers, or web links for publicly available datasets
- A description of any restrictions on data availability
- For clinical datasets or third party data, please ensure that the statement adheres to our policy

We plan to upload all data on th Figshare repository upon acceptance. If this a problem, please let me know.

Field-specific reporting

Please select the one below that is the best fit for your research. If you are not sure, read the appropriate sections before making your selection.

☒ Life sciences ☐ Behavioural & social sciences ☐ Ecological, evolutionary & environmental sciences

For a reference copy of the document with all sections, see nature.com/documents/nr-reporting-summary-flat.pdf

Life sciences study design

All studies must disclose on these points even when the disclosure is negative.

Sample size

The programs G*Power (18) from the University of Düsseldorf and the free statistical software R (19) were used to calculate the effect sizes and animal numbers. The calculation was based on the estimated effect sizes of all assessed parameters. Therefore, parameters with repeated measures (e.g., burrowing, body weight) required a "repeated measures between factors" design to differentiate statistically between the four groups. Additionally, within-between interactions and within factors were analyzed whenever appropriate. The "Cohen 1988" setting (80% power and α=5%) was used to calculate the effect size, not the usual setting in G*Power. The parameter with the largest sample size was body weight (f=0.4) in the within-between interaction with n=112. To determine the sample size for a multicenter study, the intra-cluster correlation coefficient (ICC) was used. Therefore, a realistic ICC of rho=0.09 was assumed to calculate the sample size between the laboratories. Each laboratory effectively required n=61.15 --> 62 animals. The experiments were to be conducted in group housing with 4 animals per cage, resulting in an adequate sample size of n=64 per laboratory site. (see statistical analyses)

Data exclusions

The experimental unit was the individual animal for all individually assessed parameters (burrowing, open field, elevated o-maze, stress coping test, body weight, wound score, fur score, and whisker score). For cage-based behaviors or physiological parameters, the experimental unit was the cage (nesting, fecal corticosterone metabolites, aggression). We excluded one mouse due to medical problems on site 3 and excluded a group cage from all parameters except aggression wound, fur, and whisker score. (see statistical analyses)

Replication

Multicentric study with fixed, harmonized and non-harmonized factors simultaneously in 4 different lab sites. The outcomes were directly reported.

Randomization

At each site, the animals were randomly allocated to single or group housing upon arrival and remained in their condition for the project's duration. Cages were randomly distributed in the racks in the housing rooms to avoid housing all group or single-housed animals in one area or on one level. Likewise, the order of animals for behavioral tests outside the home cage was randomized each time (simple randomization; R version 4.1.156, or randomizer.org). (see Animals and housing)

Blinding

Blinding of experimenters was not possible due to housing-based evaluation (see Table 3). (see Animals and housing)

Reporting for specific materials, systems and methods

We require information from authors about some types of materials, experimental systems and methods used in many studies. Here, indicate whether each material, system or method listed is relevant to your study. If you are not sure if a list item applies to your research, read the appropriate section before selecting a response.

Materials & experimental systems

n/a	Involved in the study	
☒	☐	Antibodies
☒	☐	Eukaryotic cell lines
☒	☐	Palaeontology and archaeology
☐	☒	Animals and other organisms
☒	☐	Human research participants
☒	☐	Clinical data
☒	☐	Dual use research of concern

Methods

n/a	Involved in the study	
☒	☐	ChIP-seq
☒	☐	Flow cytometry
☒	☐	MRI-based neuroimaging

Animals and other organisms

Policy information about studies involving animals; ARRIVE guidelines recommended for reporting animal research

Laboratory animals

Each laboratory received 32 male and 32 female C57Bl/6NRj mice at the age of 5 weeks from Janvier Labs (Le Genest-Saint-Isle, France) in a coordinated delivery: mice from the same litters were distributed to different locations. (see Animals and housing)

Wild animals

not applicable

Field-collected samples

not applicable

Ethics oversight

The experiments were approved by the respective local authorities according to the German Animal Welfare Protection Law (LANUV

Ethics oversight	A: AZ81-02.04.2020.A377; Regierungspräsidium Karlsruhe HD: 35-9185-81-G69-20 and MA 35-9185-81-G81-19; government of Upper Bavaria MÜ: ROB-55.2-2532.Vet_02-19-31). The testing sites were alphabetized as CIMH Mannheim, IBF Heidelberg, LMU Munich, and RWTH Aachen (Institute for Experimental Molecular Imaging). According to the ARRIVE guidelines 2.0 (11) the 3R principles and the Basel declaration were considered for all investigations. (see Ethical statements)

Note that full information on the approval of the study protocol must also be provided in the manuscript.
